# Estimating the causal effect of cardiometabolic conditions on socioeconomic and healthcare outcomes: a scoping review of Mendelian randomization studies

**DOI:** 10.1186/s13561-026-00724-0

**Published:** 2026-02-02

**Authors:** Sara Pedron, Xiao Tan, Juliane Maushagen, Anna-Janina Stephan, Jacob Burns, Eleanor Sanderson, Kaitlin Wade, Michael Laxy

**Affiliations:** 1 Professorship of Public Health and Prevention, School of Medicine and Health, Technical University of Munich, Munich, 80992 Germany; 2 Institute for Medical Informatics, Biometry and Epidemiology, Ludwig-Maximilians-Universität München, Munich, 81377 Germany; 3 Population Health Sciences, Bristol Medical School, University of Bristol, Beacon House, Queens Road, Bristol, BS8 1QU UK; 4 Medical Research Council Integrative Epidemiology Unit (MRC-IEU), Oakfield House, Oakfield Grove, Bristol, BS8 2BN UK

**Keywords:** Cardiometabolic conditions, Socioeconomic status, Healthcare cost, Health utility, Instrumental variables, Mendelian randomization

## Abstract

**Background:**

Cardiometabolic risk factors and conditions are the leading contributors to morbidity and mortality, yet quantifying their causal effects on socioeconomic outcomes using observational data is challenging due to endogeneity. Using genetic variants as instrumental variables, Mendelian randomization (MR) offers a unique approach to strengthen causal inference in this context and has also gained popularity in health economic literature.

**Aims:**

This study aimed to: i) map the current landscape of MR studies evaluating the impact of cardiometabolic exposures on healthcare and socioeconomic outcomes; ii) describe how core MR assumptions were tested and reported; iii) summarize how additional assumptions underlying causal interpretation were discussed.

**Methods:**

We searched MEDLINE and EMBASE for studies applying MR to examine the impact of cardiometabolic risk factors or conditions (e.g., obesity, blood pressure, cholesterol, coronary artery disease, type 2 diabetes) on socioeconomic and healthcare outcomes (e.g., education, income, occupational status, social deprivation, healthcare use and costs, health-related quality of life). Study characteristics, MR design choices, and reporting of assumption testing and causal interpretation were extracted and narratively summarized.

**Results:**

Sixteen studies were included, covering 79 exposure–outcome pairs. Most studies examined the effects of body mass index on employment or healthcare costs. Only one study assessed home ownership, social income transfers, resource utilization, and quality-adjusted life years as outcomes, respectively. Effects of childhood cardiometabolic exposures were rarely examined beyond educational outcomes. UK Biobank was the predominant data source. None of the core MR assumptions were mentioned across all studies. While weak instrument bias was frequently tested, less than 40% of studies assessed associations between instruments and observable confounders as falsification tests. Only few studies discussed monotonicity or homogeneity assumptions.

**Conclusions:**

Although MR is a promising identification strategy for assessing causal effects of cardiometabolic risk on healthcare and socioeconomic outcomes, reporting practices for assumption testing and causal interpretation vary widely. This review highlights opportunities to strengthen transparency and coherence in future MR applications. With increasing data availability and clearer methodological guidance, MR could complement conventional observational approaches in supporting policy decisions.

**Supplementary Information:**

The online version contains supplementary material available at 10.1186/s13561-026-00724-0.

## Introduction

Cardiovascular and metabolic conditions (CMD), including coronary artery disease, myocardial infarction, stroke, and type 2 diabetes, are among the most important drivers of morbidity and mortality globally [1]. Risk factors for these conditions include excess body weight, high blood pressure, and high cholesterol, among others [2–4]. Individuals with these health conditions may suffer various morbidity consequences and are in need of constant disease (self-) management. These can have serious repercussions on an individual’s ability to work and increase the need for healthcare utilization [5–7]. Consequently, these conditions and their risk factors can lead to decreased quality of life and increased healthcare costs, but also to lower productivity and higher absenteeism and therefore to lower employment and career chances [8–11]. Health policies tackling CMD and its risk factors are therefore necessary to reduce the associated health and economic burden.

However, policy impact evaluation relies on valid estimates of downstream consequences caused by the disease. Quantifying the causal effect of CMD and its risk factors on healthcare and socioeconomic outcomes is particularly challenging due to trait complexity and endogeneity in observational data, which introduces substantial uncertainty, especially when informing long-term policy evaluations and economic modeling.

Recent developments in genetic epidemiology [12–14] have enabled the wider use of individual genotypes as exogenous sources of variation in modifiable health conditions, including cardiometabolic risk factors and diseases. This method, called Mendelian randomization (MR), exploits the random variation in an individual’s genetic endowment. Concretely, MR uses single nucleotide polymorphisms (SNPs) that are associated with the exposure of interest as instruments [15, 16]. By design, MR can mitigate biases from e.g., unobserved confounders, reverse causation, and measurement errors that commonly affect observational studies, thereby strengthening causal inference.

Although MR was originally developed and widely applied in epidemiology to identify disease risk factors and therapeutic targets, its use has increasingly extended to health economics.^1^ The growing availability of genotype data linked to large epidemiologic cohorts and administrative health records has facilitated its application to investigate the causal effects of medical risk factors and conditions on downstream socioeconomic and healthcare outcomes [17, 18]. As a result, MR has emerged as a promising approach for addressing research questions highly relevant in health economics and policy evaluation.

However, the validity of causal inference from MR studies relies on specific assumptions, some of which cannot be empirically tested (see Sect. "Principles of Mendelian randomization"). Assessing how MR assumptions are reported and evaluated is thus important for both conceptual and practical reasons. From a conceptual perspective, violations or inadequate consideration of MR assumptions undermine the respective causal claims. From a practical perspective, insufficient assessment of MR assumptions increases the risk of biased estimates and their misinterpretation in the published literature, with direct implications for evidence synthesis and meta-analyses, and even policy evaluations, since many of the analyzed exposure-outcome associations in this review are important parameters for model-based economic evaluations of preventive or therapeutic interventions targeting cardiometabolic diseases.

With the growing interest in applying MR in health economics, alongside the rapid development of alternative estimation methods and sensitivity analyses and given that causal effects derived from MR may differ conceptually from those obtained using conventional methodologies, a systematic overview of current methodological practices is warranted. Such an overview can help identify common approaches, gaps and sources of inconsistency, and improve transparency and interpretation of MR-based evidence in this field.

The aim of this scoping review is to map and describe the implementation of MR in estimating the effect of cardiometabolic conditions or risk factors on individual socioeconomic and healthcare outcomes. Specifically, we aim to:

1. map the current landscape of existing MR studies examining these causal relationships to facilitate identification of potential research gaps for those interested in expanding on this evidence base in future studies;
2. describe current practices regarding reporting, testing, and where relevant, mitigation strategies for the violations of the core MR assumptions; and
3. summarize how authors discussed and causally interpreted their estimates by taking into account additional MR assumptions.

## Principles of Mendelian randomization

The principles of MR have been extensively described in recent guidelines [19] and reviews [20–23]. Here, we provide a brief overview of the rationale, assumptions, and estimation methods of MR and refer to previous research and Additional file 1: Appendix 1 for further explanations.

Conducting an observational study to infer causality merits attention due to its inherent problem of endogeneity, which can arise through three channels: reverse causation (e.g., employment chances and occupational level might determine the mental and physical health of an individual), unobserved confounding (e.g., personal ambition and motivation may be drivers of educational and employment choices as well as lifestyle), and measurement errors (e.g., self-reported weight and height may differ from the true BMI). Previous studies have implemented IV method to address endogeneity, with biological relatives’ phenotypes being commonly utilized as instruments [24–26]. While informative, residual bias (e.g., shared family environment as an unobserved confounder) cannot be entirely ruled out.

MR is a special form of IV analysis that exploits genetic variation, most commonly SNPs, as an instrument for the proposed exposure to estimate causal effects. According to Mendel's laws of segregation and independent assortment, SNPs are randomly inherited at conception and are transmitted independently of environmental, developmental, and other external factors confounding observational studies, which facilitates their use as valid instruments in MR contexts [27, 28].

As with every IV approach, MR estimates rely on plausible and valid instruments. The three core MR assumptions and further conditions necessary for causal interpretation of the estimated effects are summarized in Table 1 and presented in more detail in Additional file 1: Appendix 1. Briefly, the core assumptions assess the presence of a causal effect and require that the genetic instrument should: (1) be correlated with the exposure; (2) not share any common cause of the outcome; and (3) can only impact the outcome via the exposure of interest. Further relevant conditions, including homogeneity, monotonicity, and stable unit treatment value (SUTVA) assumption, otherwise termed “point-estimate-identifying conditions” [20], are crucial to determining the magnitude of the MR estimates. These conditions are also necessary to determine whether these can be interpreted as a local average treatment effect (LATE), under monotonicity, or as average causal effect (ACE), under homogeneity or SUTVA. Except for the relevance assumption, all other MR assumptions cannot be empirically verified and require assumption-specific falsification tests and subject-matter knowledge for their justification.

**Table 1:** MR assumptions and selected potential solutions

*Part I. Core assumptions for testing instrument validity and presence of a causal effect*			
Assumptions	Description	Violation and consequences	Potential solutions by violation*
A1 Relevance assumption [29, 30]	The genetic instrument is related to the exposure	Violation: low correlation/explanatory power of SNPs with the exposure (weak instrument bias) Consequence: can bias the estimates towards the observational ones in MR1 and towards the null in MR2 studies	Select genome-wide significant SNPs that have been investigated in replication studies Using genetic risk score instead of single SNP
A2 Independence assumption [29, 30]**	Random assignment of the genetic instrument: The instrument is not correlated with any confounder of the instrument-outcome relation	Violation: residual confounding due to population stratification, assortative mating, dynastic effects, linkage disequilibrium, etc Consequence: can bias the effect estimate in either direction	Control for observable confounders Control for principal components to ensure the homogeneity of the study population Conduct within-family analyses
A3 Exclusion restriction [29, 30]**	The impact of the genetic instrument on the outcome is entirely mediated by the exposure	Violation: alternative paths from the instrument to the outcome through linkage disequilibrium and horizontal pleiotropy Consequence: can bias the effect estimate in either direction	Scan and exclude SNPs in linkage disequilibrium and/or have pleiotropic effects Conduct pleiotropy-robust MR analyses

*Part II. Further assumptions and conditions for causal effect interpretation*			
Assumptions	Description	Interpretation of MR estimates	
Homogeneity [31, 32]	The instrument-exposure association or the effect of the exposure on the outcome is the same in the entire population	Interpreted as an average causal effect for the entire population Weaker assumptions, such as no effect modification assumption [33] and no simultaneous heterogeneity assumption [34] are also sufficient to assume homogeneity	
Monotonicity [32, 35]	The genetic instrument has a monotonic relationship to the exposure	Interpreted as a local average treatment effect for the complier subgroup, i.e., individuals whose exposure status changes in the same direction as the change of their genetic instruments Identification of the complier subgroup can be difficult	
Stable unit treatment value assumption (SUTVA) [30, 36]	No hidden variations of the treatment: there are no different versions of each treatment level across individuals No interference: the potential outcome for each individual is independent of the exposure of other individuals	Interpreted as a causal effect if variation in the exposure induced by genetic variants has the same effect on outcomes as variations in the exposure induced by lifestyle or environmental factors, which is also known as “gene-environment equivalence” in MR context	
Time horizon: genetic variants are fixed throughout the entire life course, and MR estimates can generally be interpreted as the lifetime effect of exposure on the outcome. [27]. Specifically, if the SNP-exposure association is constant over the lifespan, this lifetime effect can be understood as the effect of having a genetic liability to the exposure that results in a one-unit higher level of exposure across the life course [37]. If the SNP-exposure association is time-varying (e.g., the genetic impact of being exposed to higher BMI differs in childhood and adulthood), MR estimates can be interpreted as a lifetime effect of being on the path of the exposure associated with having a one-unit higher level of exposure at the time it is measured [20, 38]			

^*^The list of potential solutions to assumption violation is non-exhaustive. For a more comprehensive overview of potential solutions, see [39] and [40]

^**^The cited definition presents the independence assumption and the exclusion restriction as distinct yet complementary conditions: the independence assumption is a statistical condition, while the exclusion restriction is a structural one. However, any violation of the exclusion restriction inherently results in a violation of the independence assumption [36, 41, 42]

In terms of study types, MR can be designed as a *one-sample* MR (MR1), in which individual data on SNP, exposure and outcome are drawn from the same sample, or a *two-sample* MR (MR2), in which summary statistics on the SNP-exposure and SNP-outcome associations come from two independent samples, which should represent the same population (or have similar SNP-exposure associations) and should not overlap [43]. Summary data can either be taken from available genome-wide association studies (GWASs) or computed using available individual data [44]. When multiple SNPs are used as instruments, two-stage least squares (2SLS) regression can be applied for MR1, or ratio estimators for each SNP can be meta-analyzed using the inverse-variance weighted (IVW) method for MR2. Multiple SNPs can also be combined into a single genetic risk score (GRS) based on the number of risk-increasing alleles. In this case, Wald ratio estimators can be computed for both MR1 and MR2 [23, 29]. Detailed information on MR estimation methods and MR2-specific assumptions is available in Additional file 1: Appendix 1. In addition, novel methods have been developed that are less reliant on strong model assumptions and more robust in the presence of weak instruments, including likelihood-based estimators, Bayesian methods, and semiparametric methods, each with its own set of properties and limitations [23, 40, 45]. Therefore, it has been advocated using the most strict method (e.g., IVW) as the main analysis and employing a series of sensitivity analyses using alternative methods, the results of which should then be compared across all methods to demonstrate and enhance the consistency and reliability of MR estimates, while at the same time addressing issues of multiple testing [20, 21].

## Methods

We conducted a scoping review to address our study aims. Scoping reviews are designed to map the breadth of existing literature, clarify key concepts, and identify evidence gaps, and are therefore well suited to exploratory research questions in areas with emerging evidence [46]. Following the framework from Arksey and O´Malley [47] and the PRISMA-ScR reporting guidelines [48], this scoping review is structured around five stages [47]: (1) identifying the research question, (2) identifying relevant studies, (3) selecting studies, (4) charting the data, and (5) collating, summarizing and reporting the results.

The protocol was registered and is publicly available on the Open Science Framework (https://osf.io/jt98e/) before Stage 2 started. A PRISMA-ScR checklist is provided in Additional file 1: Appendix 2.

Given that our original search was conducted on 14 June 2022 and to ensure that our pool of included studies is up to date, we conducted a targeted search update from 15 June 2022 onwards. This targeted approach comprised searching only MEDLINE instead of the full set of databases considered in the original search. We decided on this targeted approach as all studies included in the original review were indexed in MEDLINE.

### Stage 1: identifying the research question

The present scoping review aims to map and summarize available studies assessing the effect of one or more cardiometabolic risk factors or conditions on any individual socioeconomic, cost or productivity, or health utility outcome using MR techniques.

We defined cardiometabolic conditions as any chronic noncommunicable disease of the cardiovascular system (e.g., coronary heart disease, myocardial infarction, and stroke) or type 2 diabetes, i.e., a cluster of very prevalent illnesses that share common modifiable metabolic risk factors [49]. Several conditions, including congenital cardiovascular abnormalities, infections of the cardiovascular system, carcinomas, pregnancy-related cardiovascular complications, gestational diabetes, and type 1 diabetes, do not fall under this definition.

We defined relevant risk factors as all metabolic and circulatory parameters that are known to affect the pathogenesis of the targeted cardiometabolic conditions. These include, for example, body mass index (BMI) or body weight, systolic blood pressure (SBP), fasting blood sugar, total cholesterol/HDL- and LDL-cholesterol fractions (henceforth “cholesterol”), and triglyceride levels. We considered all single continuous parameters (e.g., BMI or SBP), the presence of the respective health conditions (e.g., overweight or hypertension), the presence of a combination of risk factors (i.e., metabolic syndrome), and the presence of atherosclerosis as relevant risk factors.

Our outcomes consisted of individual socioeconomic status, cost or productivity indicators, and health utility outcomes. Specifically, we considered any outcome defining the social and/or economic status of an individual, such as education, occupational status and position, income, prestige, deprivation, labor market participation, early retirement, disability pension, etc. Among the cost and productivity outcomes, we considered any indicator of direct cost, indirect cost, productivity in monetary units (healthcare costs, indirect costs, etc.), or other units (healthcare utilization, sickness days, presentism, etc.) at the individual level. Regarding health utility, we included any generic index or profile score that is directly or indirectly used to compute utility values (e.g., EQ-5D, health utility index, SF12, WHO well-being index, etc.).

We abstained from a more specific definition of these outcomes to incorporate a wide range of indicators and methodological and theoretical approaches to studying the social and economic consequences of cardiometabolic diseases.

Finally, we focused on studies that investigated these relationships using an MR approach, i.e., studies that make use of the variation in an individual’s genetic endowment as an IV to assess the causal effect of an exposure (in our case, cardiometabolic risk factors or conditions) on an outcome (in our case, any individual socioeconomic, cost/productivity, or health utility outcome) [15]. We considered any type of MR (one-sample, two-sample, univariable, multivariable, etc.) or generally any IV study employing one or several SNPs or a GRS as instruments.

### Stage 2: identifying relevant studies

We systematically retrieved studies by searching MEDLINE and EMBASE, both via Ovid. Our search strategy consisted of a combination of search terms for the exposures (cardio-metabolic risk factors and conditions) and for the specific MR methodology (the complete search strategy is available in Additional file 1: Appendix 3).

We purposely did not include any keywords for the defined outcomes of interest to identify the broad range of indicators in scope. The fit of each paper with the defined outcomes was then evaluated during the screening phase. We included only studies with human subjects; we did not restrict the search to any time limit (inception to 14 June 2022; updated 17 December 2025), and we only considered studies with English full text.

Furthermore, to identify studies potentially missed by our search strategy, we applied forward (in Scopus, last searched on the 26 July 2022) and backward citation searching (reference lists) to all articles included after full-text screening.

### Stage 3: study selection

All retrieved studies were screened and selected according to pre-specified eligibility criteria (Table 2) as defined in *Stage 1*. Only studies that conducted MR analysis as their main method to answer the research question were included (i.e., studies that implemented MR analyses as an intermediate step, for example, to gather information for modeling and simulation, were not considered). Likewise, studies that specifically aimed to present or test new methodologies, which provided examples that included our exposures and outcomes of interest, were excluded.

**Table 2:** Inclusion and exclusion criteria

	Inclusion criteria	Exclusion criteria
Population	All ancestries and geographical locations, all age ranges	
Exposure	One or more cardio-metabolic diseases or risk factors	Congenital cardiovascular abnormalities, cardiovascular infections, carcinomas, adverse cardiovascular pregnancy consequences, type 1 diabetes, gestational diabetes
Control	(Implicit: defined by analysis type)	
Outcome	Any type of individual socioeconomic, healthcare, or health utility outcome	
Analysis type	Any MR analysis, i.e., any study using genetic variants as instrumental variable	Studies using non-genetic variables or indirect genetic proxies as instrumental variables (e.g., illness or risk status of family members)
Types of studies included	Original research studies that carried out the MR as main goal of the study	Reviews, protocols, etc

After applying our search strategy to the two selected databases, we retrieved all the articles from EndNote and eliminated any duplicates. Subsequently, the studies were transferred to Rayyan [50], where two independent researchers (SP and JM) screened all the retrieved articles and made a first selection of studies based on titles and abstracts. At this stage, we only excluded studies that were clearly irrelevant. Discrepancies were discussed in the group to reach a consensus (SP, JM, AJS, JB, ML).

All articles identified as potentially relevant during title and abstract screening underwent full-text screening. Initially, one reviewer (SP) screened each full text. Subsequently, the second reviewer (JM) duplicated the screening of all studies excluded by the first reviewer to ensure that no relevant studies were incorrectly excluded. For all studies excluded at this stage, we recorded the reason for exclusion.

The same procedure was applied to studies retrieved via forward–backward citation search.

### Stage 4: charting the data/data extraction

After identifying all the articles that met the inclusion criteria, we extracted all the relevant information into an a priori-defined data extraction template. We extracted general information from the studies and information on the exposure(s), outcome(s), estimation method(s), and sensitivity analyses. For brevity, different measures for education (i.e., years of education, school absence, exam results, highest educational attainment, and degree-level education), employment (i.e., years of employment, current employment status, job class, and weekly working hours), and healthcare costs (i.e., annual healthcare costs, inpatient costs, and outpatient costs) were grouped together. Furthermore, we recorded whether assumptions were mentioned, how they were discussed and tested, and which solutions were adopted in case of potential violations. Finally, we retrieved information on the results as well as their discussion and interpretation. Slight changes to the initial template were implemented to allow a more precise extraction of information on assumptions, discussion, and results.

### Stage 5: collating, summarizing and reporting the results

To narratively summarize the retrieved information, we conducted basic descriptive analyses of the extent, nature, and distribution of the included studies. We then summarized whether the three core assumptions were discussed and tested, which tests were used, and which solutions were adopted in case of assumption violation. Furthermore, where available, we reported discussions and tests of further MR assumptions and analyzed and reported any discussions regarding the validity, interpretation, and generalizability of the estimates.

As a scoping review, this study aimed to describe how MR is applied and reported in health economics research, rather than to formally evaluate the methodological quality of individual studies. Consistent with established guidance for scoping reviews [47, 48] and in the absence of a consensus risk of bias tool for MR studies [51, 52], a formal quality appraisal was not undertaken.

## Results

### Overview of retrieved studies

**Fig. 1:**
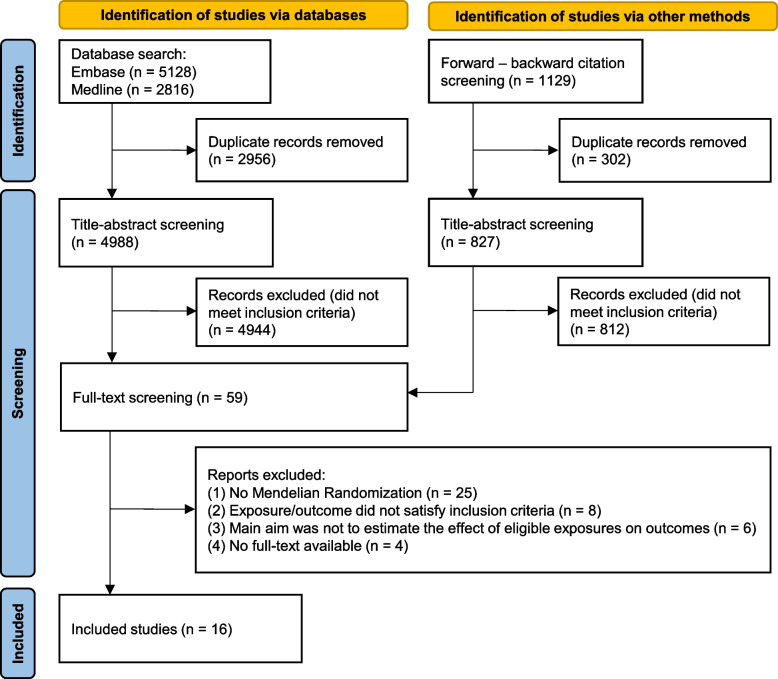
PRISMA flow diagram

The initial search yielded 4988 unique studies after deduplication. Another 827 studies were identified via forward–backward screening of references and citations of included studies. After title and abstract screening, 59 articles remained for full-text screening. Of these, 43 did not meet our inclusion criteria; the reasons for exclusion are recorded in the flow diagram (Fig. 1) and detailed in the full-text screening table (Additional file 1: Appendix 4).

Following the screening process, 16 studies met the eligibility criteria and were included in the narrative synthesis [17, 18, 53–66]. The updated search in MEDLINE did not identify any additional eligible studies.

### Narrative synthesis of included studies

**Fig. 2:**
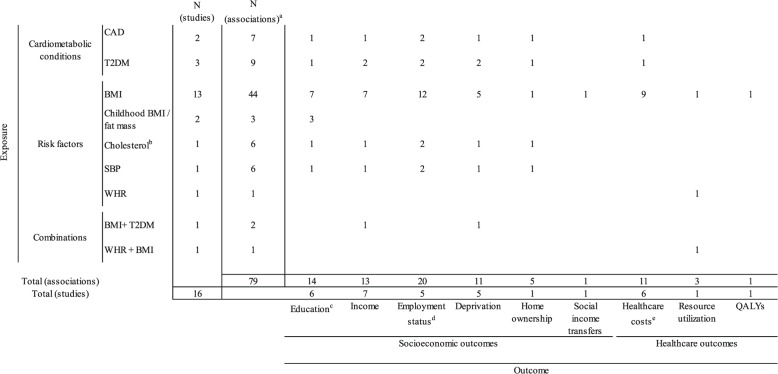
Exposures and outcomes investigated in the included studies. Each distinct exposure-outcome association investigated in the main MR analysis was counted. Abbreviations: *BMI* body mass index, *CAD* coronary artery disease, *QALYs* quality-adjusted life years, *SBP* systolic blood pressure, *SES* socioeconomic status, *T2DM* type 2 diabetes mellitus, *WHR* waist-hip ratio. ^a^Number of associations refers to the number of exposure-outcome associations estimated in each study (i.e. sum of the table rows). ^b^Cholesterol refers to total cholesterol or HDL-and LDL-fractions. ^c^Education-related outcomes include degree-level education, highest educational attainment, and years of education for adult population, and exam scores and school absence for children. ^d^Employment status includes years of being employed, weekly working hours, job class (skilled vs. unskilled), and current employment status. ^e^Healthcare costs include total annual healthcare costs, inpatient costs, and outpatient costs

The number of exposures, outcomes, and their associations analyzed in each study are displayed in Fig. 2. (Detailed information is available in Additional file 2: Appendix 5) The most frequently investigated outcomes were indicators of individual socioeconomic status, including education [56–58, 60, 64, 65], income [18, 53, 54, 56–58, 60], employment status [53, 56–58, 60], and regional deprivation [18, 56–58, 60]. Other studies also investigated other socioeconomic indicators such as home ownership [56] and receipt of social income transfers [53]. Among healthcare outcomes, healthcare costs were investigated in six studies [17, 55, 61–63, 66], whereas only one study [59] reported on resource utilization and one study [63] on quality of life.

With regard to exposures, 13 out of 16 studies investigated BMI [17, 18, 53–63], followed by type 2 diabetes [18, 56, 66], coronary artery disease [56, 66], and childhood BMI/fat mass [64, 65]. Further exposures studied were cholesterol and systolic blood pressure [56], waist-hip ratio [59], and combinations such as BMI controlling for type 2 diabetes [18] and waist-hip ratio controlling for BMI [59].

For the 16 included studies, a total of 79 exposure-outcome associations were reported, with the effect of BMI on employment status being investigated the most. Notably, except for BMI and waist-hip ratio, no study estimated the impact of other cardiometabolic risk factors on healthcare costs, utilization, or quality of life. Furthermore, no study investigated the relationship between childhood BMI or fat mass and healthcare outcomes.

Regarding the type of MR employed, the majority of studies [54–56, 59, 61, 63, 64, 66] coupled an MR1 analysis with sensitivity analyses using MR2 methods. Five studies conducted only an MR1 [53, 58, 60, 62, 65] and three studies conducted only an MR2 analysis [17, 18, 57]. (Details are available in Additional file 2: Appendix 5).

**Fig. 3:**
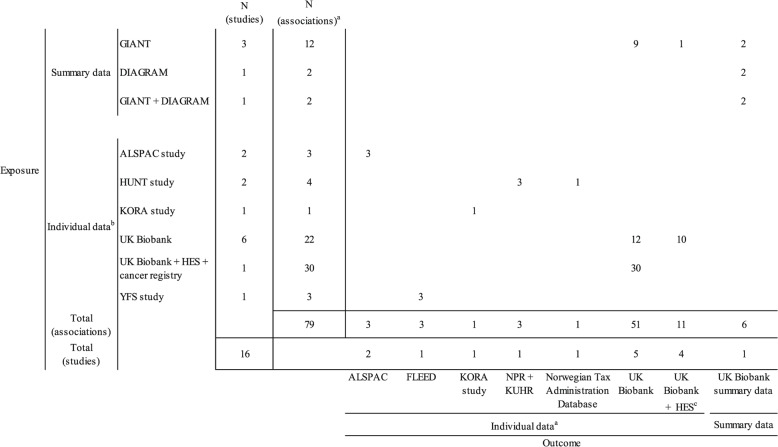
Data sources used in the included studies. Only data sources used for the reported main MR analysis were considered. Abbreviations: *ALSPAC* Avon Longitudinal Study of Parents and Children, *DIAGRAM* Diabetes Genetics Replication and Meta-analysis, *FLEED* Finnish Longitudinal Employer-Employee Data, *GIANT* Genetic Investigation of Anthropometric Traits, *HES* Health Episode Statistics, *HUNT* Nord-Trøndelag Health Studies, *KORA* Cooperative Health Research in the Augsburg Region, *KUHR* Norwegian Control and Distribution of Health Reimbursement Database, *NPR* Norwegian Patient Register, *YFS* Young Finns Study. ^a^Number of associations refers to the number of exposure-outcome associations estimated in each study. ^b^Individual data were used either as disaggregated data for MR1 analysis or were computed to summary statistics for MR2 analysis

Figure 3 provides an overview of the data sources used in the main analysis of the included studies. (see Additional file 2: Appendix 5 for detailed information) For studies conducting an MR1 analysis, individual-level data from UK Biobank for both SNP-exposure and SNP-outcome associations were most frequently utilized [56, 58–60, 62, 63, 66], which is often linked with Health Episode Statistics (HES) [56, 59, 62, 63, 66]. Other studies used regional data sources for both exposure and outcome measurements, including the Avon Longitudinal Study of Parents and Children (ALSPAC) study in England [64, 65], the Cooperative Health Research in the Region Augsburg (KORA) study in Germany [61], the Nord-Trøndelag Health Studies (HUNT) study in Norway [54, 55], and the Young Finns Study (YES) Study in Finland [53].

Among the three studies carrying out exclusively MR2 analyses [17, 18, 57], all used summary data from available GWASs [67, 68] for SNP-exposure associations. For the SNP-outcome associations, two studies computed summary statistics either using available UK Biobank individual data alone [57] or linked with HES data [17]. One study used publicly available summary statistics based on UK Biobank participants from the MRC Integrative Epidemiology Unit (IEU) UK Biobank GWAS pipeline [18, 69].

### Results summary

Table 3 depicts the effect direction of the associations investigated in the included studies using an MR approach. A clear negative effect of cardiometabolic risk factors and conditions on education, income, being employed, and home ownership, and a clear positive effect on deprivation level and healthcare costs were found in most of the included studies. In contrast, evidence on social income transfers, resource utilization, and QALYs was scarce and was only researched within the context of BMI and waist-hip ratio [53, 59, 63]. Similarly, the impacts of coronary artery disease, type 2 diabetes, cholesterol, and systolic blood pressure on socioeconomic outcomes were mostly investigated in a single study [56], which did not reveal any strong associations. Overall, MR estimates were partly robust to sensitivity analyses. Inconsistent results were frequently observed when MR2 pleiotropy-robust methods (especially MR-Egger) or within-family analyses were applied. These pleiotropy-robust methods have lower statistical power and precision given the same sample size and within-family analyses are usually based on much smaller study samples.

**Table 3:** Effect direction plot for the MR estimates from the main analysis of the 16 included studies

Exposure	CAD	˄[56]	˅[56]	˄[56] ˄[56]	˄[56]	˅[56]		▲ [66]		
	T2DM	˅[56]	˅[56]˅[18]	˄[56] ˅[56]	˄[56]˄[18]	˅[56]		˄ [66]		
	BMI	˄[57] ▼[56]▼[58]▼[58] ▼[58] ˅[60]˅[60]	**▼**[53]**▼**[56]**▼**[57]**▼**[58]**▼**[18]**▼**[60] ˅[54]	▲[56]▲[57] ˄[57]˄[57]˄[57] ▼[53]▼[56]▼[58] ▼[57] ˅[57]˅[58]˅[60]	▲[56]▲[58]▲[18]▲[60] ▼[57]	▼[56]	˄[53]	▲[55]▲[61]▲[62] ▲[17]▲[63]▲[63] ▲[63] ˄[55]˄[55]	▲[59]	▼[63]
	Childhood BMI/fat mass	▲[64] ▼[64]˅[65]								
	Cholesterol*	˅[56]	˄[56]	˄[56]˄[56]	˄[56]	˄[56]				
	SBP	˄[56]	˅[56]	˄[56] ˅[56]	˄[56]	˅[56]				
	WHR								˄[59]	
	BMI + T2DM		˅[18]		˄[18]					
	WHR + BMI								▲[59]	
		Education-related outcomes	Income	Employment status	Deprivation	Home ownership	Social income transfers	Healthcare costs	Resource utilization	QALYs
		* Socioeconomic outcomes*						* Healthcare outcomes*		
	Outcome									

*Abbreviations*: *BMI* body mass index, *CAD* coronary artery disease, *GRS* genetic risk score, *IVW* inverse-weighted method, *QALYs* quality-adjusted life years, *SBP* systolic blood pressure, *SES* socioeconomic status, *SNP* single-nucleotide polymorphism, *T2DM* type 2 diabetes mellitus, *WHR* waist-hip ratio

Effect directions were based on associations estimated (N= 79) in the main analysis of the 16 included studies. As three studies [18, 55, 58] did not explicitly indicate their main analyses, we reported their effect estimates according to the most frequently applied methods (e.g., IVW as main estimator in MR2, GRS instead of single SNP as instrument, and using the full set of covariates) from other included studies. ▲positive significant, ▼negative significant, ˄ positive insignificant, ˅ negative insignificant. Statistical significance based on threshold chosen in each study (usually at a p-value of 0.05, lower thresholds are possible when adjustment for multiple testing was conducted)

^*^Cholesterol refers to total cholesterol or HDL- and LDL-cholesterol fractions

Explanation on effect direction of each outcome category:

• Education-related outcomes include degree-level education, highest educational attainment, years of education for adult population, and exam scores and school absence for children. A positive effect means that an increase in exposure leads to a higher education level, more years of education, higher exam scores, and a lower possibility of school absence. Negative effect means an increase in exposure will lead to a lower education level, fewer years of education, lower exam scores, and a higher possibility of school absence

• A positive (negative) effect of exposure on income means that an increase in exposure leads to higher (lower) income

• Employment status includes working hours/years, job class (skilled vs. unskilled) and current employment status (not in paid employment, sick/disabled, caring for home/family, retired and unemployed) in comparison with employed individuals. In general, a positive effect means that an increase in exposure leads to more working hours/years, higher odds of having a skilled job and lower odds of being employed. A negative effect means that an increase in exposure leads to lower working hours/years, a lower odd of having a skilled job, and higher odds of being employed. One study [58] estimated odds ratio of being employed with unemployed, meaning that an increase (decrease) in exposure results in higher (lower) odds of being employed

• A positive (negative) effect of exposure on deprivation means that an increase in exposure leads to higher (lower) deprivation level

• A positive (negative) effect of exposure on home ownership means that an increase in exposure leads to higher (lower) chance of owning a home

• A positive (negative) effect of exposure on social income transfers means that an increase in exposure leads to higher (lower) chance of receiving social income transfers

• Healthcare costs include total annual healthcare costs, inpatient costs, and outpatient costs. A positive (negative) effect of exposure on healthcare costs means that an increase in exposure leads to higher (lower) healthcare costs

• A positive (negative) effect of exposure on resource utilization means that an increase in exposure leads to higher (lower) health service usage

• A positive (negative) effect of exposure on QALYs means that an increase in exposure leads to higher (lower) quality of life

With one exception [18], all included studies qualitatively compared the effect magnitude and direction of MR estimates with those of conventional regression estimates (mostly using ordinary least squares method, OLS). For BMI and waist-hip ratio, the effect directions across both MR and conventional regression analyses were comparable, but MR estimates tended to be lower for socioeconomic outcomes and slightly larger for healthcare costs. One study [56] assessed the impact of SBP, cholesterol, coronary heart disease, and type 2 diabetes on a range of socioeconomic outcomes using MR and OLS methods. Regardless of the statistical method, the effect of SBP remained consistent, but the OLS estimates of cholesterol were larger and demonstrated a positive association with all socioeconomic outcomes. The results also found insignificant effects of coronary heart disease and type 2 diabetes when using MR, but greater effect estimates when using OLS. For healthcare costs, one study [66] reported a greater positive effect of coronary artery disease and type 2 diabetes estimated by MR than by OLS. The MR result estimates and a comparison with non-MR models of each study are documented in Additional file 2: Appendix 6.

### The three core MR assumptions

We summarized the assumption tests and discussions in Table 4, 5, 6 and 7 and provided the corresponding data of each included study in Additional file 2: Appendix 7. The STROBE-MR reporting guidelines [70] for MR studies define explicit mentioning and discussions of all three MR assumptions as quality criteria for MR studies. Table 3 shows that the three core assumptions were explicitly mentioned and defined in most but not all studies. Specifically, the relevance and independence assumption were mentioned in 56% and 63% of the studies, respectively, whereas the exclusion restriction was mentioned in 88% of all the included papers.

**Table 4:** Summary of assumptions mentioned

	Total		MR1^a^		MR1 & MR2		MR2	
	16		5		8		3	
*Core assumptions*								
Explicit statement of relevance assumption	9	56%	3	60%	4	50%	2	67%
Explicit statement of independence assumption	10	63%	3	60%	5	63%	2	67%
Explicit statement of exclusion restriction	14	88%	4	80%	7	88%	3	100%
*Further assumptions*								
Mentioning homogeneity	2	13%	0	0%	1	11%	1	33%
Mentioning monotonicity	4	25%	0	0%	3	38%	1	33%
Mentioning SUTVA	11	69%	2	40%	7	88%	2	67%

Full results per study can be found in Additional file 2: Appendix 7**.** We deemed this criterion fulfilled when the authors explicitly mentioned the assumption and briefly described it

*Abbreviations*:* MR1* studies that carried out only one-sample MR, *MR1* & *MR2* studies that carried out both one-sample and two-sample analysis, *MR2* studies that carried out only two-sample analysis, *SUTVA,* stable unit treatment value assumption

^a^Two studies [58, 60] were categorized as MR1 because of a limited description and result reporting of MR2 as sensitivity analysis in the main paper

**Table 5:** Discussion, tests, and solutions adopted for violations of relevance assumption (weak instrument bias)

	Total		MR1^b^		MR1 & MR2		MR2	
	16		5		8		3	
Explicitly mentioning weak instruments bias/its consequences	10	63%	3	60%	5	63%	2	67%
*Tests*								
Proportion of variance explained by SNPs/GRS (R2/pseudo R2 in own study or original GWAS)	11	69%	4	80%	5	63%	2	67%
First-stage association between SNPs/GRS and exposure + p-value	8	50%	2	40%	4	50%	2	67%
First-stage F-statistic > 10 (and p-value)	14	88%	4	80%	8	100%	2	67%
Comparison of main MR estimates with other methods robust to weak instruments (e.g., MR-RAPS)	3	19%	0	0%	1	13%	2	67%
Other^a^:								
- Correlation between above-average GRS and exposure to test the relevance of the instruments								
- Comparing own results with estimation using a larger GRS which explains 5% of total variation in BMI								
- Reporting first-stage Kleibergen-Paap Wald rk F-statistic								
- Reporting conditional Sanderson-Windmeijer F-statistic for multivariable MR1 analyses								
- Comparison of results with main GRS and with LASSO selected GRS								
- Comparison of predicted BMI per unit score increase with the score of original GWAS								
*Solutions*								
Choosing only GWAS significant SNPs	16	100%	5	100%	8	100%	3	100%
Use of GRS^c^	14	88%	4	80%	8	100%	2	67%
Other*:								
- Use of a larger SNP in sensitivity analysis that explains 5% of the variation in exposure								
- Split-sample GWAS using UK Biobank data								
- Excluding genotypes with lower probability and poor imputation quality (both < 0.9)								
- Excluding variants that did not reach GWAS significance in all strata, or if they were classified as secondary signals within one locus								
- LASSO variable selection								
- MR2: MR-RAPS method^d^								

Full results for each study are available in Additional file 2: Appendix 7

*Abbreviations*: *MR* Mendelian randomization, *MR1* studies that carried out only one-sample MR, *MR1* & *MR2* studies that carried out both one-sample and two-sample analysis, *MR2* studies that carried out only two-sample analysis, *BMI* body mass index, *GRS* genetic risk score, *GWAS* genome-wide association studies, *LASSO* least absolute shrinkage and selection operator, MR-*RAPS* MR using a robust adjusted profile score, *SNPs* single nucleotide polymorphisms

^a^ “Other” includes tests and solutions that were only applied by one study. The corresponding studies can be found in Additional file 2: Appendix 7

^b^Two studies [58, 60] were categorized as MR1 because of a limited description and result reporting of MR2 as sensitivity analysis in the main paper

^c^The use of GRS is a common solution to weak instrument bias but it was also used by some authors [63] to reduce bias from single pleiotropic SNPs

^d^MR-RAPS is a technique that can be used to account for weak instrument bias and pleiotropy. One study [18] applied this method to obtain pleiotropy-robust estimates as shown in Table 6 and Additional file 2: Appendix 7

**Table 6:** Discussion, tests, and solutions adopted for violations of independence assumption (confounding)

	Total		MR1^b^		MR1 & MR2			MR2
	16		5		8			3
Mentioning random allocation at conception	15	94%	5	100%	7	88%	3	100%
Mentioning confounding via pre-existing factors (year of birth, time of conception, etc.)	6	38%	2	40%	3	38%	1	33%
Confounding of SNP-outcome associations:								
Population stratification/ancestral differences	14	88%	4	80%	7	88%	3	100%
Assortative mating	11	69%	3	60%	7	88%	1	33%
Dynastic effect bias	11	69%	2	40%	6	75%	3	100%
Geographical stratification	5	31%	2	40%	2	25%	1	33%
Collider bias	4	25%	1	20%	1	13%	2	67%
Cohort effects	3	19%	0	0%	2	25%	1	33%
Tests								
(Jointly) testing association between SNPs/GRS and covariates that might impact outcome	6	38%	4	80%	1	13%	1	33%
(Qualitative) Comparison of main MR estimates with estimates from sensitivity analyses	9	56%	3	60%	5	63%	1	33%
Solutions								
Adjustment for principal components	11	69%	2	40%	7	88%	2	67%
Adjustment for potential confounders	15	94%	5	100%	8	100%	2	67%
Controlling for parental characteristics (to control for assortative mating)	3	19%	3	60%	0	0%	0	0%
Using ethnically homogeneous population	11	69%	4	80%	6	75%	1	33%
Applying within-family/within-sibling analysis	5	31%	1	20%	3	38%	1	33%
Exclusion of individuals who were genetically over-related	8	50%	2	40%	4	50%	2	67%
Exclusion of SNPs that are not in Hardy–Weinberg equilibrium	4	25%	2	40%	1	13%	1	33%
Conducting split-sample analysis (to ensure the homogeneity of the GWAS and the study population)	2	13%	0	0%	2	25%	0	0%
Other^a^:								
- Comparison of main MR analysis with non-genetic analyses								
- Comparison of stratified analysis using Fisher’s Z-score method								
- BOLT-LMM approach to confirm that results were robust to potential influence of population stratification								
- Converting data of three traits to normal distribution to reduce bias from population stratification								
- Residualizing exposure and outcome variables in standard instrumental variable method to address population stratification								
- Additional exclusion of SNPs based on Information Content and Minor Allele Frequency Criteria								
- Use of F-statistic to compare stratified analysis for age groups and main MR analysis								
- Restriction to individuals of white European ancestry for exposure in a sensitivity analysis								

Full results for each study available in Additional file 2: Appendix 7

*Abbreviations*: *MR* Mendelian randomization, *MR1* studies that carried out only one-sample MR, *MR1* & *MR2* studies that carried out both one-sample and two-sample analysis, MR2 studies that carried out only two-sample analysis, *SNPs* single nucleotide polymorphisms, *GRS* genetic risk score, *GWAS* genome-wide association studies, *BOLT*-*LMM* Bayesian mixed model algorithm

^a^“Other” includes tests and solutions that were only applied by one study each. The corresponding studies can be found in Additional file 2: Appendix 7

^b^Two studies [58, 60] were categorized as MR1 because of a limited description and result reporting of MR2 as sensitivity analysis in the main paper

**Table 7:** Discussion, tests, and solutions adopted for violations of assumption 3 (pleiotropy and linkage disequilibrium)

		Total	MR1^c^		MR1&MR2		MR2	
		16	5		8		3	
Mentioning linkage disequilibrium	8	50%	2	40%	4	50%	2	67%
Mentioning pleiotropy	16	100%	5	100%	8	100%	3	100%
*Tests*^*a*^								
MR1: Sargan´s overidentification test^*a*^	3	23%	2	40%	1	13%	n.r.	n.r.
MR2: intercept of MR-Egger regression	11	100%	n.r.	n.r.	8	100%	3	100%
MR2: Cochran`s Q statistic (assuming balanced heterogeneity)	9	82%	n.r.	n.r.	6	75%	3	100%
MR2: Rücker´s Q statistic (assuming unbalanced heterogeneity)	2	18%	n.r.	n.r.	1	13%	1	33%
MR2: I2GX (regression dilution statistic)	4	36%	n.r.	n.r.	2	25%	2	67%
MR2: leave-one-out analysis (forest plot)^*a*^	3	27%	n.r.	n.r.	2	25%	1	33%
MR2: single SNPs effects (forest plot)^*a*^	7	64%	n.r.	n.r.	5	63%	2	67%
Scatterplots of SNP-outcome/SNP-exposure association (for testing heterogeneity/outliers)	5	31%	1	20%	2	25%	2	67%
Graphical analysis of results from main and sensitivity analyses to indicate invalid instruments (using funnel plots, forest plots, qualitative comparison, etc.)^a^	13	81%	2	40%	8	100%	3	100%
Other^b^:								
- Estimation of the correlation between each disease-specific GRSs in both the main analyses and within each split in the split-sample analyses, to determine whether any of the GRSs share genetic information								
- Using RadialMR plots to identify outliers								
- Conducting gene-by-environment interaction to detect and correct for pleiotropy								
*Solutions*^*a*^								
MR2: pleiotropy-robust estimates^*a*^								
MR-Egger	11	100%	n.r.	n.r.	8	100%	3	100%
Penalized/weighted/simple median MR	11	100%	n.r.	n.r.	8	100%	3	100%
Weighted/simple modal MR	11	100%	n.r.	n.r.	8	100%	3	100%
MR-Egger with SIMEX correction	2	18%	n.r.	n.r.	2	25%	0	0%
sisVIVE	1	9%	n.r.	n.r.	1	13%	0	0%
Multivariable MR	3	19%	0	0%	1	13%	2	67%
Investigating and excluding SNPs with known pleiotropic effects (literature/NGHRI GWAS catalog)	5	31%	5	100%	0	0%	0	0%
Investigating and excluding SNPs with known pleiotropic effects (Phenoscanner)	4	25%	0	0%	3	38%	1	33%
Generating a reduced GRS with evidence for less pleiotropy (own study)	2	13%	1	20%	1	13%	0	0%
Searching and excluding SNPs in linkage disequilibrium/selecting independent SNPs	10	63%	2	40%	5	63%	3	100%
Investigating and excluding outliers	6	38%	1	20%	5	63%	0	0%
Other^b^:								
- Comparison of main MR analysis with non-genetic analyses								
- Comparison between genetic and non-genetic IVs								
- Performing MR-RAPS to model balanced pleiotropy								
- Conducting gene-by-environment interaction to detect and correct for pleiotropy								

The frequency for the total number of studies was adjusted by the number of studies for which the respective test or solution was deemed relevant (i.e., for tests or solutions that are carried out in the context of an MR2 analysis, we excluded MR1 studies in the final count). Full results for each study available in Additional file 2: Appendix 7

*Abbreviations*: *MR* Mendelian randomization, *MR1* studies that carried out only one-sample MR, *MR1* & *MR2* studies that carried out both one-sample and two-sample analysis, *MR2* studies that carried out only two-sample analysis, *BMI* body mass index, *GRS* genetic risk score, *GWAS* genome-wide association studies, *IV* instrumental variables, *NGHRI* National Human Genome Research Institute, *n.r. *not relevant, *SIMEX* simulation extrapolation procedure, *sisVIVE* Some Invalid Some Valid Instrumental Variable Estimation, *SNPs* single nucleotide polymorphisms

^a^All tests and solutions adopted serve as indicators of validity of instruments, against violations of independence and exclusion restriction. However, most studies included recognized pleiotropy as the most salient threat to validity. Therefore, tests and solutions for the validity of instruments are reported here and interpreted by the authors are violations due to pleiotropy or solutions against pleiotropy

^b^“Other” includes tests and solutions that were only applied by one study (except for the solution MR2 robust estimates using sisVIVE). The corresponding studies can be found in Additional file 2: Appendix 7

^c^Two studies [58, 60] were categorized as MR1 because of a limited description and result reporting of MR2 as sensitivity analysis in the main paper

#### Weak instrument bias (violations of relevance assumption)

Table 5 displays a summary of discussion, tests, and solutions adopted for violations of the relevance assumption. Weak instrument bias was explicitly mentioned in 63% of the studies. The most frequently used test for this assumption was the first-stage F-statistic based on the threshold of 10 suggested by Staiger and Stock (1997) [71], which was reported in 14 studies (88%). Furthermore, 69% of the included studies reported a measure of the variance explained by the instruments, either from the original GWASs or computed within their own dataset. An additional test is to analyze the magnitude and significance of the first-stage coefficient(s), which was carried out in 50% of the studies. Further tests include variations of the F-statistic (e.g., Kleibergen-Paap Wald rk F-statistic, conditional Sanderson-Windmeijer F-statistic) or comparisons of the estimates from the main MR analysis with the results from estimation methods that are robust to weak instruments (e.g., MR-RAPS estimation).

All studies selected only GWAS-significant SNPs to reduce the possibility of weak instrument bias. Another solution was the use of a GRS to reduce concerns related to single/multiple variants being weak instruments. Among all included studies, 88% used a GRS as an instrument alongside investigating models with single SNPs in sensitivity analyses. One study [61] explicitly stated that using a GRS can also reduce the likelihood of pleiotropy induced by any single SNP and therefore increase instrument validity. Additionally, two studies employed weak-instrument robust methods, such as MR-RAPS estimation [17] and LASSO variable selection [61].

#### Confounding (violations of independence assumption)

Table 6 shows the practices and discussion related to confounding in the included studies. Overall, 15 studies (94%) mentioned that conditional on parental genotype, random allocation at conception ensured a random distribution of variants, from which six studies (38%) also mentioned the possibility of confounding by preexisting factors at conception. All studies reported one or more types of confounding of the instrument/exposure-outcome associations. Concretely, 88% mentioned population stratification, 69% assortative mating, 69% dynastic effect bias, 31% geographic stratification, and 27% collider bias as important issues in their analyses. Three studies also considered cohort effects as another source of confounding.

To test the violations of the independence assumption, six studies (38%) investigated the association between the instrument(s) (single SNPs or GRS) and observed covariates. Furthermore, nine studies (56%) compared the estimates from the primary analysis with estimates from more robust alternative estimation methods in sensitivity analyses, such as within-family analysis or analysis using non-genetic instruments.

The most frequent solutions to counteract residual confounding were adjustment for observable confounders (94%), adjustment for principal components (69%), and focusing on an ethnically homogeneous population for both exposure and outcome data (69%). Moreover, half of the studies excluded genetically over-related individuals, while 31% performed within-family or within-siblings analyses. Three studies controlled for parental characteristics to address potential assortative mating (19%). Finally, four studies (25%) explicitly excluded SNPs that were not in Hardy–Weinberg equilibrium and another two studies (13%) performed a split-sample analysis to ensure homogeneity of the exposure and outcome populations.

#### Pleiotropy and linkage disequilibrium (violations of exclusion restriction)

As shown in Table 7, all included studies mentioned and discussed pleiotropy, while only eight (50%) mentioned linkage disequilibrium. Note that, although the presented tests and solutions can also be applied for the independence assumption, we reported them in the context of exclusion restriction as most studies recognized pleiotropy as the greatest threat to instrument validity and performed tests explicitly for pleiotropy.

The validity of the instruments was tested using Sargan’s overidentification test in MR1 investigations (3 out of 13 studies, 23%) and Cochran’s Q (9 out of 12 studies, 75%) or Rücker’s Q statistic (2 out of 12 studies, 17%) in MR2 investigations. The MR-Egger intercept test was investigated in all MR2 studies as an indicator of pleiotropy.

Further tests were conducted in MR2 analyses to examine heterogeneity and/or MR2-related assumptions. Commonly used tests for SNP heterogeneity include visualization of the estimated results using single SNPs (*n* = 7, 64%) and of those from leave-one-out analyses (*n=*3, 27%). The regression dilution statistic (I2GX, *n=*4, 36%) was also computed to detect measurement error in the SNP-exposure association.

Finally, five studies (31%) used scatter plots depicting the SNP-outcome against the SNP-exposure association to identify heterogeneous effects as an indicator for violation of independence or exclusion restriction assumptions. Thirteen studies (81%) compared results from the main MR analysis to those from sensitivity analyses employing pleiotropy-robust methods. Qualitative differences in the effect estimates were interpreted as indicators of invalid instruments, mostly because of pleiotropy.

These pleiotropy-robust methods typically use summary data, which include MR-Egger (*n=*11, 100%), median- and mode-based estimators (*n=*11, 100%), MR-Egger with a SIMEX (*n=*2, 18%), and sisVIVE estimation (*n=*1, 9%). Further solutions adopted to address violations of exclusion restriction include performing a multivariable two-sample MR (*n=*3, 19%), investigating and excluding SNPs with known pleiotropic effects based on available literature (*n=*5, 31%) or Phenoscanner (*n=*4, 25%), generating a GRS excluding SNPs that had evidence for pleiotropy (*n=*2, 13%), excluding SNPs in linkage disequilibrium (*n=*10, 56%), and removing outliers (*n=*6, 38%).

### Further assumptions for causal interpretation

As summarized in Table 3, four studies (25%) mentioned and briefly discussed monotonicity [17, 59, 63, 66]. They explicitly stated that, although untestable, monotonicity was a reasonable assumption in their context. One of these studies cited the original GWAS paper for the instrument-exposure relations [17, 67], stating that monotonicity was biologically plausible and that no evidence for opposite effects in different subgroups was evident. Together with three other studies [53, 56, 61], these studies interpreted the estimated results as local average treatment effects (LATEs). From them, two studies [17, 66] expanded upon monotonicity and specified that given the continuous exposure variable, the complier subgroup identified by monotonicity is likely to represent the entire target population, indicating that the homogeneity assumption would be satisfied.

Furthermore, 11 studies [17, 53–59, 61, 63, 66] mentioned directly or indirectly the SUTVA, stating that the estimated effects correspond to the effects generated by a genetically induced lifetime exposure to the risk factor, which might cause different effects compared to exposures affected by environmental exposures (e.g., parental socioeconomic status) or interventions. For this reason, the authors advised caution when generalizing the results to other contexts.

### Generalizability and main residual concerns

Regarding the generalizability of MR estimates, interpretations differ depending on the dataset utilized. Authors using data from UK Biobank [17, 56, 57, 59, 62, 63, 66] or ALSPAC [64, 65] studies stated that these data were not representative of the whole UK population, so the generalizability of their results was limited. In contrast, authors using data from Norwegian registries [55] stated that their data were representative of the whole population, allowing for generalization for the country. Other mentioned issues hampering generalizability include a strictly homogeneous population of white European descent [58, 64], restricted age groups [60], or restricted geographical areas investigated [53, 57].

Further cautions in the interpretation of the results were discussed. First, despite the abovementioned attempts to test and address potential violations of the three core assumptions, several authors [18, 53–55, 62, 64, 65] recognized identifying pleiotropy and the lack of in-depth knowledge of the role and function of single SNPs and pathways as a major challenge. Second, the nonlinear relationship between BMI and outcomes, which would affect the correct interpretation of the results if ignored, was investigated in seven studies (38%) [17, 54, 55, 58, 61–63]. Third, among the 11 studies that performed MR2, seven studies (44%) [17, 18, 56, 57, 63, 64, 66] reported using two different samples for exposure and outcome estimates to avoid biases caused by sample overlap. Fourth, some authors [56, 60] stated that since no bidirectional analysis was carried out, reverse causation could not be ruled out. Finally, many authors [17, 55, 56, 59–61, 63–66] have discussed lower statistical power as a limitation, with four studies [55, 60, 61, 64] also conducting empirical power calculations. Together, they warned that null effects should be interpreted as no evidence of an effect rather than evidence of no effects. A detailed report on each study regarding further discussion can be found in Additional file 2: Appendix 6 and 7.

## Discussion

In this scoping review, we included 16 studies investigating the effect of cardiometabolic risk factors and conditions on individual socioeconomic and healthcare outcomes using Mendelian randomization (MR). Our first objective was to map the existing evidence and identify potential gaps. We found that most of the included studies focused on BMI, with other conditions and risk factors, such as cardiovascular disease, type 2 diabetes, cholesterol, and blood pressure being more sparsely investigated. Furthermore, we found no studies investigating the effects of other cardiometabolic disorders (e.g., myocardial infarction, stroke, or angina) despite the well-documented correlations with direct and indirect costs reported in previous research [10, 72, 73].

There are several feasibility-related explanations for the concentration on BMI and the relative scarce evidence for other cardiometabolic conditions despite their high disease burden and policy relevance, illustrating how data availability and instrument characteristics shape the current MR evidence base. First, among the cardiometabolic conditions examined in this review, BMI is one of the variables with the greatest variance explained by genetic instruments (2.7% based on 97 loci, as reported in the GWAS study [67] that was mostly used by the included studies) because it does not require a longitudinal cohort or a case–control study to measure its occurrence and its association with genetic variants, facilitating the development of GWASs with larger study samples to assess these associations (e.g., up to 339,224 participants in [67]). Furthermore, in contrast to outcomes such as high blood pressure, type 2 diabetes, or coronary heart disease, for which effective medical treatments exist and the endpoint measurement might be biased, BMI can be measured more easily and accurately without increasing the risk of selection bias and survival bias. However, even for BMI, the explained variance is very low, requiring a much larger sample size to ensure sufficient explanatory power and prediction precision, which might also explain the rather scarce evidence available for other conditions. Nevertheless, the low variance explained by genetic variants does not necessarily preclude the use of these variants as valid instruments, providing that they are strongly associated with the exposure. Nowadays, larger GWAS studies are increasingly available, which explain greater proportions of variance in cardiometabolic diseases and conditions [74, 75]. This opens new opportunities for researchers to study new associations by means of more robust genetic instruments for other phenotypes beyond BMI. Another reason for the large number of MR studies on BMI can be that for acute events, such as myocardial infarction and stroke, alternative estimation strategies, such as panel regression fixed effect models, might be more suitable than IV methodologies, although previous studies have estimated the impact of those acute events on other outcomes using MR techniques [76, 77].

A wide array of individual socioeconomic and health outcomes was investigated; however, the bulk of the evidence focuses on the effect of BMI on socioeconomic indicators and healthcare costs, while healthcare utilization and QALYs were investigated in only one study each (Table 4). Some of the studies on healthcare costs were able to link genetic data with survey data or health statistics taken from official sources [17, 55, 59, 63, 66], so the respective outcome variables are less prone to recall bias than self-reported socioeconomic outcomes. Future research should establish similar linkages with national employment and/or retirement databases to obtain more robust sources of information on these outcomes.

Most studies drew their information on exposure and/or outcome from UK Biobank data [17, 18, 56–60, 62, 63, 66], which represents an important limitation of the available evidence. In fact, previous studies [78, 79] have raised concerns about the representativeness of the UK Biobank for the UK population, and thus, most studies in our review are likely to be affected by collider bias or other types of selection bias [80, 81]. For example, participants in the UK Biobank are healthier, more affluent, and better educated than the general population and can therefore introduce spurious correlation between exposure (e.g., BMI) and outcome (e.g., employment status) [57, 79]. Furthermore, the predominant use of UK Biobank results for both exposures and outcomes (“sample overlap”) causes serious problems to the causal interpretation of results due to correlated errors, type 1 error inflation, and violation of the independence assumption, particularly in the presence of weak instruments [43, 82, 83].

The strong reliance on UK Biobank reflects a broader structural constraint: few alternative data sources currently combine genetic information with large-scale linkable survey or administrative data with relevant healthcare and socioeconomic outcomes suitable for MR analyses. Although recent GWAS have substantially expanded locus discovery for cardiovascular diseases [74, 84] and increasingly include multiple ancestries [85], translation into MR studies of policy-relevant outcomes is limited by the availability of genotyped outcome data, sufficiently powered outcome GWAS, and the ability to minimize bias from sample overlap and population stratification. Nonetheless, emerging population-based datasets and biobanks (such as lifelines [86], the NAKO study [87], or the CONSTANCES study [88]), illustrate potential pathways for future research. As data access, linkage, and harmonization improve, these resources may enable the investigation of a broader range of cardiometabolic exposures. Moreover, expanding GWAS and linked outcome data beyond white European populations will be particularly important for improving representativeness and generalizability of MR studies.

Our second objective was to analyze discussions, tests, and solutions adopted for the three core MR assumptions in the included articles. According to the STROBE-MR reporting guidelines [70], the authors of MR studies should explicitly state the core assumptions (item 5), describe the methods or prior knowledge used to assess the assumptions (item 7), and report the assessment of the validity of the assumptions and any additional statistics (item 12). Inadequate assessment of the core assumptions increases the risk of biased estimates, while insufficient reporting limits transparency and reproducibility [21], hindering the appropriate use of MR findings in evidence synthesis, policy, and economic modeling. We found that whereas the exclusion restriction was explicitly mentioned in most studies (88%), the relevance and independence assumptions were mentioned in only 56% and 63% of the studies, respectively (Table 4). These results indicate that researchers might be more concerned with the exclusion restriction assumption, but not enough attention was devoted to potential problems with weak instruments (violation of relevance assumption) and confounding (violations of independence assumption). Therefore, we advocate for a more comprehensive and structured definition and discussion of all three core IV assumptions in MR studies as suggested by the STROBE-MR guidelines [70].

According to item 7 and 12 of the STROBE-MR guidelines [70], all studies carried out, where possible, at least basic tests to assess the assumptions and adopted solutions to counteract potential problems. Here, we briefly discuss the major findings from this large summary of methods. First, our analysis showed that most studies estimated and reported the first-stage F-statistic as an indicator of weak instrument bias using a threshold of 10 [71]. Noting that this threshold is a proxy of the true critical value chosen based on a specific, yet not widely understood definition of weak instrument, which requires homoscedastic serially uncorrelated error terms [89], alternative definitions and tests of weak instruments would lead to a higher F-statistic threshold [90, 91]. Depending on the research purpose (i.e., minimizing bias vs. hypothesis testing) and data features (i.e., heteroscedasticity, autocorrelation, and clustering), it might be helpful to consider other thresholds and robustness tests [91, 92]. To address possible weak instrument bias, all studies selected only SNPs that reach genome-wide significance of *P* < 5.0 × 10^–8^. However, it should be noted that this significance level by no means ensures the relevance of the instruments, which is dependent on sample size and can be altered by lower-frequency alleles and higher linkage disequilibrium thresholds [93], indicating the need for a more stringent definition of the significance level in such cases. Moreover, advanced analytical methods, including MR-cML [94], MRBEE [95] and dIVW [96], have been developed to automatically adjust for weak instruments without additional requirements of F-statistic. However, these methods often come with reduced precision in effect estimates. Thus, future studies should re-consider the context-specific thresholds of F-statistic and genome-wide significance and exploit the novel approaches alongside conventional MR methods to control for weak instrument bias.

Second, our review showed a lower use of falsification tests to assess independence assumption and exclusion restriction as well as inconsistent approaches to identifying and adjusting for confounding. In fact, only six out of 16 studies (38%) tested the correlation of the instrument with observable, mostly post-conception confounders. Since this is one of the few falsification tests that can be performed for the independence assumption or the exclusion restriction [97], not conducting these tests is a lost opportunity to falsify the assumptions or to strengthen the arguments around the validity of the instruments. The most commonly used strategy in case of violation due to confounding is adjustment for observable covariates of the exposure–outcome associations. Although 75% of the studies adjusted for observable confounders, only a few have correctly stressed that including too many covariates in MR could induce collider bias, because these are phenotypes that can be affected by genetic instruments and outcomes.

In addition, several studies did not explicitly report strategies to limit genetic relatedness between participants (50%) or to account for heterogenous ancestry groups (30%). This may reflect implicit assumptions based on e.g., established biobank recruitment practices, GWAS quality-control procedures, or domain knowledge about cohort composition, rather than these issues being ignored in practice. More generally, this highlights a broader challenge in evaluating how single studies addressed MR assumptions, as insufficient reporting may or may not indicate insufficient or inadequate assumption assessment that can impact causal estimates. Nonetheless, limited transparency would restrict readers’ ability to assess potential bias, evaluate instrument validity and transportability, and compare findings across studies.

Furthermore, issues regarding nonlinearity and statistical power were investigated by several authors. Seven studies [17, 54, 55, 58, 61–63] employed the residualization method [98, 99] to examine the nonlinear pattern of BMI and outcomes. This approach estimates the stratum-specific effect by creating and conditional on the so-called “IV-free” exposure (which equals the first-stage error term) and can avoid collider bias from directly stratifying on the exposure [98, 99]. Recent research [100–102], however, indicates that this approach could generate spurious estimates and induce considerable bias, as it requires a linear and constant effect of the instrument on the exposure in each stratum, which may not hold in many cases. The doubly ranked method [103], a novel development of the residualization method, also failed to yield plausible results despite being designed to be less sensitive to the constant genetic effect assumption [100, 101]. Therefore, it is advised that further application of these nonlinearity methods should be halted until stronger evidence regarding their reliability is available [100]. Moreover, only four studies [55, 60, 61, 64] conducted empirical power calculations. Despite the growing availability of GWAS summary-level statistics and large-scale epidemiological studies with genotype information, the variance in traits explained by genetics remains small, indicating the necessity for a large sample size to increase statistical power and prediction precision. A-priori power calculation can therefore be helpful for identifying and selecting the most suitable dataset and should be incorporated into planning an MR study [19].

Our third objective was to summarize the discussion around causal interpretation of the findings. We found that monotonicity was only mentioned by four studies [17, 59, 63, 66] and only one [17] tried to provide some evidence to support the plausibility of monotonicity in the investigated context. Although untestable, the monotonicity assumption is essential for interpreting the magnitude of MR estimates: if monotonicity does not hold, the point estimate of MR is not interpretable. When monotonicity holds, MR estimates are to be interpreted as LATE, making it critical to identify the complier subgroup for which the MR results can be applied [35]. Two [17, 66] of the four studies which mentioned monotonicity further stated that the estimated LATE could be interpreted as an average causal effect (ACE) for the entire target population because of the continuous exposure variable, implicitly suggesting the fulfillment of the homogeneity assumption. One possible explanation for the low reporting of monotonicity could be an insufficient awareness of its role in causal interpretation. Alternatively, many studies may implicitly assume effect homogeneity and therefore interpret MR estimates as population average effects without explicitly addressing monotonicity or the distinction between LATE and ACE.

Furthermore, eleven studies [17, 53–59, 61, 63, 66] directly or indirectly mentioned the SUTVA assumption. Generally, the no hidden variations of the treatment assumption (Table 1), which coincides with the gene-environment equivalence assumption in genetic epidemiology, is more questionable, as it is uncertain whether variation in the exposure induced by SNPs has the same effect on outcomes as variations in the exposure induced by lifestyle or environmental factors. This assumption is complicated because MR estimates capture the effects of a genetically induced lifetime exposure to cardiometabolic risk factors and not to instantaneous or time-defined changes in cardiometabolic risk factors (e.g., through policy intervention). Hence, caution is warranted when using MR estimates for modeling purposes, where a differentiated use of estimates from various sources is needed. Given the overall low rate of studies reporting these assumptions, our findings suggest that researchers need to be more sensitive to a thorough description, investigation, and evaluation of the assumptions crucial to causal interpretation.

Additionally, our study revealed interesting insight into the differences between MR and OLS results. Even though the two methods appear to estimate the same effects, they differ substantially: OLS estimates the association between the exposure status and the outcome at a specific time and is subject to bias arising from endogeneity, whereas MR estimates the causal effect of lifetime exposure to risk factors due to genetic predisposition and is less prone to bias from reverse causation and confounding. Therefore, comparisons of the magnitude and direction of estimates from both methodologies should be interpreted with these differences in mind. Nevertheless, such comparisons can still shed light on the likely direction of bias and the potential influence of other factors (e.g., socioeconomic factors, environmental influences, and interventions) on the exposure-outcome relationship. For the effect of BMI on socioeconomic outcomes, the magnitude of MR estimates was generally consistent with or weaker than that of OLS estimates. Conversely, studies using Scandinavian datasets, which are considered to have greater representativeness than the UK Biobank dataset, yielded stronger effect sizes using MR than using the OLS method [53, 54]. Regarding healthcare outcomes, whereas most studies demonstrated consistent or greater MR estimates, one study utilizing Scandinavian data [55] and one utilizing UK Biobank data [62] showed lower effects of BMI using MR than OLS estimates. These differences in effect estimates can be reflective of differences in the sample population, the time horizon of the estimates, or residual confounding unadjusted in OLS estimation. In all studies, the confidence intervals of MR and OLS estimates overlap, meaning that the difference in effect estimates is not significant. Although insightful, it is essential to uncover possible reasons for and underlying mechanisms of the different patterns by conducting pooled analyses using different datasets, considering contextual factors, and ensuring methodological homogeneity. Taking together, we highlight the importance of triangulating evidence from different methodologies that ideally have distinct assumptions and orthogonal sources of bias to assess and facilitate robust causal inference [20, 104].

The present scoping review is the first to provide a comprehensive overview of available MR studies on the effects of cardiometabolic conditions on socioeconomic and healthcare outcomes, with a specific focus on how the underlying assumptions are reported, assessed, and discussed. Our study complements previous research by expanding beyond BMI to include a broad range of cardiometabolic risk factors and conditions, covering diverse socioeconomic and healthcare outcomes, and by addressing aspects that are not previously considered, such as assumptions related to causal interpretation, generalization of MR estimates, and comparisons of effect direction across methods [39]. By systematically summarizing what MR assumptions are reported and how these are tested and addressed when potential violations arise, our review documents current practice in the field of health economics. This mapping provides a practical overview for researchers planning or conducting MR studies, facilitating transparency and learning across studies. It also offers an empirical foundation that may inform the development and future application of structured quality appraisal tools for MR once such instruments become available.

However, our study also has several limitations. First, since no consensus-based, validated quality appraisal checklist for MR studies is available to date, it was not possible to judge the quality of included studies against a clear gold standard. Thus, we developed our own criteria for assessing how identified studies report and discuss MR assumptions. While the development and application of such criteria are by nature subjective, we aimed to ensure that they are rigorously and consistently applied. We deemed an assumption “mentioned” only when it was explicitly referred to and briefly defined in the study; consequently, studies that discussed the implications of one assumption without explicitly mentioning or describing this assumption were considered not to fulfill this criterion. To date, several systematic reviews have summarized existing tools and checklists related to conduct, reporting, and evaluation of MR studies [51, 52]. However, also these reviews conclude that, due to limited empirical validation of identified tools and heterogeneity in assessed domains, no structured quality appraisal tool can currently be recommended for MR studies. Nonetheless, a number of registered protocols suggest that efforts are ongoing to develop validated and standardized assessment frameworks [51, 52], which may substantially improve transparency, comparability, and evidence synthesis in future MR research. Second, the lists of methods used to test and mitigate bias violating MR assumptions are based on included studies and are by no means exhaustive. They may contain more or less efficient strategies to assess and address violations of one assumption, the appropriateness of which was not assessed in this study. Thus, it is the responsibility of each researcher to compare and select the most appropriate method for the research question and data at hand. Third, as a scoping review, we did not perform a meta-analysis of the study results. However, this effort would be beneficial, especially for the effects of BMI on several frequently investigated outcomes, to determine relevant parameters for modeling studies. Finally, as our update search was conducted in MEDLINE, it is possible that a small number of eligible studies indexed elsewhere were missed; however, given the complete MEDLINE coverage of all originally included studies and the methodological focus of this review, such studies are unlikely to affect the overall conclusions of this review.

## Conclusions

This is the first scoping review that systematically summarizes the implementation of MR in examining the healthcare and socioeconomic impacts of cardiometabolic risk factors and conditions. We analyzed evidence from 16 articles that mostly covered the effect of BMI and drew on samples of European ancestry, pointing out research gaps for other cardiometabolic conditions and population subgroups. Our findings highlight the need for more transparent, context-specific reporting and critical discussion of MR assumptions, their violations, and potential mitigation strategies, especially those relevant for causal interpretation. Our study also points to the importance of effect triangulation by complementing MR with other approaches to strengthen causal inference and support policy decisions. Overall, this review synthesizes current MR practice in health economics and provides practical implications for improving transparency, interpretability, and coherence in future research.

## Supplementary Information

Additional file 1.

Additional file 2.

## Data Availability

No datasets were generated or analysed during the current study.

## Footnotes

1 We conducted a targeted PubMed search combining the term “Mendelian randomization” with keywords for key health economic outcomes, including socioeconomic status, deprivation, healthcare utilization, costs, income, education, employment, productivity, and quality of life. The search yielded in total 1116 results and showed a clear upward trend from one publication in 2006 to 342 publications in 2025. While these records were not screened for eligibility, the observed pattern illustrates the growing application of MR in health economics.

## References

[1] Vos T, Lim SS, Abbafati C, et al. Global burden of 369 diseases and injuries in 204 countries and territories, 1990–2019: a systematic analysis for the Global Burden of Disease Study 2019. Lancet. 2020;396:1204–22. 10.1016/S0140-6736(20)30925-9. 33069326 10.1016/S0140-6736(20)30925-9 PMC7567026

[2] World Health Organization (WHO). Cardiovascular diseases (CVDs). 2023. https://www.who.int/news-room/fact-sheets/detail/cardiovascular-diseases-(cvds). Accessed 6 Dec 2023.

[3] Ralston J, Nugent R. Toward a broader response to cardiometabolic disease. Nat Med. 2019;25:1644–6. 10.1038/s41591-019-0642-9. 31700172 10.1038/s41591-019-0642-9

[4] Eckel RH, Grundy SM, Zimmet PZ. The metabolic syndrome. Lancet. 2005;365:1415–28. 10.1016/S0140-6736(05)66378-7. 15836891 10.1016/S0140-6736(05)66378-7

[5] Brink E, Karlson BW, Hallberg LRM. Readjustment 5 months after a first-time myocardial infarction: reorienting the active self. J Adv Nurs. 2006;53:403–11. 10.1111/J.1365-2648.2006.03737.X. 16448483 10.1111/j.1365-2648.2006.03737.x

[6] Daniel K, Wolfe CDA, Busch MA, Mckevitt C. What are the social consequences of stroke for working-aged adults?: a systematic review. Stroke. 2009;40. 10.1161/STROKEAHA.108.534487. 10.1161/STROKEAHA.108.534487 19390074

[7] Gomersall T, Madill A, Summers LKM. A metasynthesis of the self-management of type 2 diabetes. Qual Health Res. 2011;21:853–71. 10.1177/1049732311402096. 21429946 10.1177/1049732311402096

[8] Cawley J. An economy of scales: a selective review of obesity’s economic causes, consequences, and solutions. J Health Econ. 2015;43:244–68. 10.1016/j.jhealeco.2015.03.001. 26279519 10.1016/j.jhealeco.2015.03.001

[9] Foy AJ, Mandrola JM. Heavy heart: the economic burden of heart disease in the United States now and in the future. Prim Care. 2018;45:17–24. 10.1016/J.POP.2017.11.002. 29406942 10.1016/j.pop.2017.11.002

[10] Gheorghe A, Griffiths U, Murphy A, et al. The economic burden of cardiovascular disease and hypertension in low- and middle-income countries: a systematic review. BMC Public Health. 2018;18:1–11. 10.1186/S12889-018-5806-X/TABLES/4. 10.1186/s12889-018-5806-x PMC6090747 30081871

[11] Pedron S, Emmert-Fees K, Laxy M. Schwettmann L (2019) The impact of diabetes on labour market participation: a systematic review of results and methods. BMC Public Health. 2019;19(1):1–13. 10.1186/S12889-018-6324-6. 30616606 10.1186/s12889-018-6324-6 PMC6323654

[12] Tcheandjieu C, Zhu X, Hilliard AT, et al. Large-scale genome-wide association study of coronary artery disease in genetically diverse populations. Nat Med. 2022;28:1679–92. 10.1038/s41591-022-01891-3. 35915156 10.1038/s41591-022-01891-3 PMC9419655

[13] Chen J, Spracklen CN, Marenne G, et al. The trans-ancestral genomic architecture of glycemic traits. Nat Genet. 2021;53:840–60. 10.1038/s41588-021-00852-9. 34059833 10.1038/s41588-021-00852-9 PMC7610958

[14] Hahn J, Fu Y-P, Brown MR, et al. Genetic loci associated with prevalent and incident myocardial infarction and coronary heart disease in the Cohorts for Heart and Aging Research in Genomic Epidemiology (CHARGE) Consortium. PLoS One. 2020;15:e0230035. 10.1371/journal.pone.0230035. 33186364 10.1371/journal.pone.0230035 PMC7665790

[15] Smith GD, Hemani G. Mendelian randomization: genetic anchors for causal inference in epidemiological studies. Hum Mol Genet. 2014;23.10.1093/hmg/ddu328. 10.1093/hmg/ddu328 PMC4170722 25064373

[16] Thomas DC, Conti DV. Commentary: the concept of ‘Mendelian Randomization.’ Int J Epidemiol. 2004;33:21–5. 10.1093/IJE/DYH048. 15075141 10.1093/ije/dyh048

[17] Dixon P, Hollingworth W, Harrison S, et al. Mendelian randomization analysis of the causal effect of adiposity on hospital costs. J Health Econ. 2020;70:102300. 10.1016/j.jhealeco.2020.102300. 32014825 10.1016/j.jhealeco.2020.102300 PMC7188219

[18] Pedron S, Kurz CF, Schwettmann L, Laxy M. The effect of BMI and type 2 diabetes on socioeconomic status: a two-sample multivariable Mendelian randomization study. Diabetes Care. 2021;44:850–2. 33414106 10.2337/dc20-1721 PMC7955199

[19] Burgess S, Davey Smith G, Davies NM, et al. Guidelines for performing Mendelian randomization investigations: update for summer 2023. Wellcome Open Res. 2023;4:186. 10.12688/wellcomeopenres.15555.3. 32760811 10.12688/wellcomeopenres.15555.1 PMC7384151

[20] Sanderson E, Glymour MM, Holmes MV, et al. Mendelian randomization. Nat Rev Methods Primers. 2022;2:1–21. 10.1038/s43586-021-00092-5. 10.1038/s43586-021-00092-5 PMC7614635 37325194

[21] Richmond RC, Davey Smith G. Mendelian randomization: concepts and scope. Cold Spring Harb Perspect Med. 2022;12:a040501. 10.1101/cshperspect.a040501. 34426474 10.1101/cshperspect.a040501 PMC8725623

[22] Labrecque J, Swanson SA. Understanding the assumptions underlying instrumental variable analyses: a brief review of falsification strategies and related tools. Curr Epidemiol Rep. 2018;5:214–20. 10.1007/s40471-018-0152-1. 30148040 10.1007/s40471-018-0152-1 PMC6096851

[23] Burgess S, Small DS, Thompson SG. A review of instrumental variable estimators for Mendelian randomization. Stat Methods Med Res. 2017;26:2333–55. 10.1177/0962280215597579. 26282889 10.1177/0962280215597579 PMC5642006

[24] Cawley J, Meyerhoefer C. The medical care costs of obesity: an instrumental variables approach. J Health Econ. 2012;31:219–30. 10.1016/j.jhealeco.2011.10.003. 22094013 10.1016/j.jhealeco.2011.10.003

[25] Black N, Hughes R, Jones AM. The health care costs of childhood obesity in Australia: an instrumental variables approach. Econ Hum Biol. 2018;31:1–13. 10.1016/j.ehb.2018.07.003. 30064082 10.1016/j.ehb.2018.07.003

[26] Seuring T, Goryakin Y, Suhrcke M. The impact of diabetes on employment in Mexico. Econ Hum Biol. 2015;18:85–100. 10.1016/J.EHB.2015.04.002. 25985080 10.1016/j.ehb.2015.04.002

[27] Davey Smith G, Ebrahim S. Mendelian randomization’: can genetic epidemiology contribute to understanding environmental determinants of disease? Int J Epidemiol. 2003;32:1–22. 10.1093/ije/dyg070. 12689998 10.1093/ije/dyg070

[28] von Hinke S, Davey Smith G, Lawlor DA, et al. Genetic markers as instrumental variables. J Health Econ. 2016;45:131–48. 10.1016/j.jhealeco.2015.10.007. 26614692 10.1016/j.jhealeco.2015.10.007 PMC4770870

[29] Burgess S, Thompson SG. Mendelian Randomization: Methods for Using Genetic Variants in Causal Estimation (1st ed.). Chapman and Hall/CRC. 2015.10.1201/b18084.

[30] Angrist JD, Imbens GW, Rubin DB. Identification of Causal Effects Using Instrumental Variables. Journal of the American Statistical Association. 1996;91(434),444–55. 10.1080/01621459.1996.10476902.

[31] MR Dictionary. Homogeneity assumption. 2022. https://mr-dictionary.mrcieu.ac.uk/term/homogeneity/.

[32] Hernán MA, Robins JM. Instruments for causal inference: an epidemiologist’s dream? Epidemiology. 2006;17:360–72. 10.1097/01.ede.0000222409.00878.37. 16755261 10.1097/01.ede.0000222409.00878.37

[33] MR Dictionary. No effect modification assumption (Additional IV assumption). 2022. https://mr-dictionary.mrcieu.ac.uk/term/no-effect-mod/.

[34] Hartwig FP, Wang L, Davey Smith G, Davies NM. Average causal effect estimation via instrumental variables: the no simultaneous heterogeneity assumption. Epidemiology. 2023;34:325–32. 10.1097/EDE.0000000000001596. 36709456 10.1097/EDE.0000000000001596

[35] MR Dictionary. Monotonicity assumption. 2022. https://mr-dictionary.mrcieu.ac.uk/term/monotonicity/.

[36] Imbens GW, Rubin DB. Causal inference for statistics, social, and biomedical sciences: an introduction. Cambridge: Cambridge University Press; 2015.

[37] Labrecque JA, Swanson SA. Interpretation and potential biases of Mendelian Randomization estimates with time-varying exposures. Am J Epidemiol. 2019;188:231–8. 10.1093/aje/kwy204. 30239571 10.1093/aje/kwy204

[38] Morris TT, Heron J, Sanderson ECM, et al. Interpretation of Mendelian randomization using a single measure of an exposure that varies over time. Int J Epidemiol. 2022;51:1899–909. 10.1093/ije/dyac136. 35848950 10.1093/ije/dyac136 PMC9749705

[39] Mbutiwi FIN, Dessy T, Sylvestre M-P. Mendelian randomization: a review of methods for the prevention, assessment, and discussion of pleiotropy in studies using the fat mass and obesity-associated gene as an instrument for adiposity. Front Genet. 2022;13:803238. 10.3389/fgene.2022.803238. 35186031 10.3389/fgene.2022.803238 PMC8855149

[40] Boehm FJ, Zhou X. Statistical methods for Mendelian randomization in genome-wide association studies: a review. Comput Struct Biotechnol J. 2022;20:2338–51. 10.1016/j.csbj.2022.05.015. 35615025 10.1016/j.csbj.2022.05.015 PMC9123217

[41] Angrist JD, Pischke J-S. Mostly harmless econometrics: an empiricist’s companion. Princeton: Princeton University Press; 2009.

[42] Wooldridge JM. Econometric analysis of cross section and panel data. 2nd ed. Cambridge, Massachusetts London, England: MIT Press; 2010.

[43] Burgess S, Davies NM, Thompson SG. Bias due to participant overlap in two-sample Mendelian randomization. Genet Epidemiol. 2016;40:597–608. 10.1002/gepi.21998. 27625185 10.1002/gepi.21998 PMC5082560

[44] Davies NM, Holmes MV, Davey Smith G. Reading Mendelian randomisation studies: a guide, glossary, and checklist for clinicians. BMJ. 2018;362:601. 10.1136/BMJ.K601. 10.1136/bmj.k601 PMC6041728 30002074

[45] Davies NM, von Hinke Kessler Scholder S, Farbmacher H, et al. The many weak instruments problem and Mendelian randomization. Stat Med. 2015;34(3):454–68. 10.1002/sim.6358. 25382280 10.1002/sim.6358 PMC4305205

[46] Munn Z, Peters MDJ, Stern C, et al. Systematic review or scoping review? Guidance for authors when choosing between a systematic or scoping review approach. BMC Med Res Methodol. 2018;18:143. 10.1186/s12874-018-0611-x. 30453902 10.1186/s12874-018-0611-x PMC6245623

[47] Arksey H, O’Malley L. Scoping studies: towards a methodological framework. Int J Soc Res Methodol. 2007;8:19–32. 10.1080/1364557032000119616.

[48] Tricco AC, Lillie E, Zarin W, et al. PRISMA extension for scoping reviews (PRISMA-ScR): checklist and explanation. Ann Intern Med. 2018;169:467–73. 10.7326/M18-0850/SUPPL_FILE/M18-0850_SUPPLEMENT.PDF. 30178033 10.7326/M18-0850

[49] Chatterjee A, Harris SB, Leiter LA, et al. Managing cardiometabolic risk in primary care: summary of the 2011 consensus statement. Can Fam Physician. 2012;58:389. 22611605 PMC3325449

[50] Ouzzani M, Hammady H, Fedorowicz Z, Elmagarmid A. Rayyan—a web and mobile app for systematic reviews. Syst Rev. 2016;5.10.1186/S13643-016-0384-4. 10.1186/s13643-016-0384-4 PMC5139140 27919275

[51] Spiga F, Gibson M, Dawson S, et al. Tools for assessing quality and risk of bias in Mendelian randomization studies: a systematic review. Int J Epidemiol. 2023;52:227–49. 10.1093/ije/dyac149. 35900265 10.1093/ije/dyac149 PMC9908059

[52] Yu J, Zou M, Spiga F, et al. Structured tools to assessing quality and bias in Mendelian randomisation studies: an updated systematic review. 2025. 2025.09.05.25335166. 10.1093/ije/dyag052 PMC13091611 41999635

[53] Böckerman P, Cawley J, Viinikainen J, et al. The effect of weight on labor market outcomes: an application of genetic instrumental variables. Health Econ. 2019;28:65–77. 10.1002/hec.3828. 30240095 10.1002/hec.3828 PMC6585973

[54] Edwards CH, Bjørngaard JH, Minet Kinge J. The relationship between body mass index and income: using genetic variants from HUNT as instrumental variables. Health Econ. 2021;30:1933–49. 10.1002/hec.4285. 33993584 10.1002/hec.4285

[55] Edwards CH, Vie GÅ, Kinge JM. Body mass index and healthcare costs: using genetic variants from the HUNT study as instrumental variables. BMC Health Serv Res. 2022;22:396. 10.1186/s12913-022-07597-z. 35337320 10.1186/s12913-022-07597-z PMC8957125

[56] Harrison S, Davies AR, Dickson M, et al. The causal effects of health conditions and risk factors on social and socioeconomic outcomes: Mendelian randomization in UK Biobank. Int J Epidemiol. 2020;49:1661–81. 10.1093/ije/dyaa114. 32808034 10.1093/ije/dyaa114 PMC7746412

[57] Campbell DD, Green M, Davies N, et al. Effects of increased body mass index on employment status: a Mendelian randomisation study. Int J Obes. 2021;45:1790–801. 10.1038/s41366-021-00846-x. 10.1038/s41366-021-00846-x PMC8310793 34158612

[58] Howe LD, Kanayalal R, Harrison S, et al. Effects of body mass index on relationship status, social contact and socio-economic position: Mendelian randomization and within-sibling study in UK Biobank. Int J Epidemiol. 2020;49:1173–84. 10.1093/ije/dyz240. 31800047 10.1093/ije/dyz240 PMC7750981

[59] Hazewinkel A-D, Richmond RC, Wade KH, Dixon P. Mendelian randomization analysis of the causal impact of body mass index and waist-hip ratio on rates of hospital admission. Econ Hum Biol. 2022;44:101088. 10.1016/j.ehb.2021.101088. 34894623 10.1016/j.ehb.2021.101088 PMC8784824

[60] Tyrrell J, Jones SE, Beaumont R, et al. Height, body mass index, and socioeconomic status: mendelian randomisation study in UK Biobank. BMJ. 2016:i582. 10.1136/bmj.i582. 10.1136/bmj.i582 PMC4783516 26956984

[61] Kurz CF, Laxy M. Application of mendelian randomization to investigate the association of body mass index with health care costs. Med Decis Making. 2020;40:156–69. 10.1177/0272989X20905809. 32154779 10.1177/0272989X20905809

[62] Dick K, Schneider JE, Briggs A, et al. Mendelian randomization: estimation of inpatient hospital costs attributable to obesity. Health Econ Rev. 2021;11:16. 10.1186/s13561-021-00314-2. 33990897 10.1186/s13561-021-00314-2 PMC8122556

[63] Harrison S, Dixon P, Jones HE, et al. Long-term cost-effectiveness of interventions for obesity: a mendelian randomisation study. PLoS Med. 2021;18:e1003725. 10.1371/journal.pmed.1003725. 34449774 10.1371/journal.pmed.1003725 PMC8437285

[64] Hughes A, Wade KH, Dickson M, et al. Common health conditions in childhood and adolescence, school absence, and educational attainment: Mendelian randomization study. NPJ Sci Learn. 2021;6:1. 10.1038/s41539-020-00080-6. 33398003 10.1038/s41539-020-00080-6 PMC7782810

[65] Von Hinke Kessler Scholder S, Davey Smith G, Lawlor DA, et al. The effect of fat mass on educational attainment: examining the sensitivity to different identification strategies. Econ Hum Biol. 2012;10:405–18. 10.1016/j.ehb.2012.04.015. 22709667 10.1016/j.ehb.2012.04.015 PMC3899051

[66] Dixon P, Harrison S, Hollingworth W, et al. Estimating the causal effect of liability to disease on healthcare costs using mendelian randomization. Econ Hum Biol. 2022;46:101154. 10.1016/j.ehb.2022.101154. 35803012 10.1016/j.ehb.2022.101154

[67] Locke AE, Kahali B, Berndt SI. Genetic studies of body mass index yield new insights for obesity biology. Nature. 2015;518:197–206. 10.1038/nature14177. 25673413 10.1038/nature14177 PMC4382211

[68] Scott RA, Scott LJ, Mägi R. An expanded genome-wide association study of type 2 diabetes in Europeans. Diabetes. 2017;66:2888–902. 10.2337/db16-1253. 28566273 10.2337/db16-1253 PMC5652602

[69] Mitchell RE, Elsworth BL, Raistrick CA, et al MRC IEU UK Biobank GWAS pipeline version 2. In: University of Bristol. https://research-information.bris.ac.uk/en/datasets/mrc-ieu-uk-biobank-gwas-pipeline-version-2. Accessed 27 July 2023.

[70] Skrivankova VW, Richmond RC, Woolf BAR, et al. Strengthening the reporting of observational studies in epidemiology using mendelian randomisation (STROBE-MR): explanation and elaboration. BMJ. 2021:n2233. 10.1136/bmj.n2233. 10.1136/bmj.n2233 PMC8546498 34702754

[71] Staiger D, Stock JH. Instrumental variables regression with weak instruments. Econometrica. 1997;65:557–86. 10.2307/2171753.

[72] Vandenberghe D, Albrecht J. The financial burden of non-communicable diseases in the European Union: a systematic review. Eur J Public Health. 2020;30:833–9. 10.1093/eurpub/ckz073. 31220862 10.1093/eurpub/ckz073

[73] Ryder S, Fox K, Rane P, et al. A systematic review of direct cardiovascular event costs: an international perspective. Pharmacoeconomics. 2019;37:895–919. 10.1007/s40273-019-00795-4. 30949988 10.1007/s40273-019-00795-4

[74] Yengo L, Sidorenko J, Kemper KE, et al. Meta-analysis of genome-wide association studies for height and body mass index in ∼700000 individuals of European ancestry. Hum Mol Genet. 2018;27:3641–9. 10.1093/hmg/ddy271. 30124842 10.1093/hmg/ddy271 PMC6488973

[75] Kettunen J, Demirkan A, Würtz P, et al. Genome-wide study for circulating metabolites identifies 62 loci and reveals novel systemic effects of LPA. Nat Commun. 2016;7:11122. 10.1038/ncomms11122. 27005778 10.1038/ncomms11122 PMC4814583

[76] Sun W, Zhang L, Liu W, et al. Stroke and myocardial infarction: a bidirectional Mendelian randomization study. Int J Gen Med. 2021;14:9537–45. 10.2147/IJGM.S337681. 34916835 10.2147/IJGM.S337681 PMC8670204

[77] Yang ZQ, et al. Causal associations between blood pressure and the risk of myocardial infarction: A bidirectional Mendelian randomization study. Frontiers in Cardiovascular Medicine 2022;9:924525. 10.3389/fcvm.2022.924525. 10.3389/fcvm.2022.924525 PMC9686852 36440027

[78] Sudlow C, Gallacher J, Allen N, et al. UK biobank: an open access resource for identifying the causes of a wide range of complex diseases of middle and old age. PLoS Med. 2015;12:e1001779. 10.1371/journal.pmed.1001779. 25826379 10.1371/journal.pmed.1001779 PMC4380465

[79] Fry A, Littlejohns TJ, Sudlow C, et al. Comparison of sociodemographic and health-related characteristics of UK Biobank participants with those of the general population. Am J Epidemiol. 2017;186:1026–34. 10.1093/aje/kwx246. 28641372 10.1093/aje/kwx246 PMC5860371

[80] Morgan J, Major-Smith D Quantifying potential selection bias in observational research: simulations and Analyses exploring religion and depression using a prospective UK cohort study (ALSPAC). Religion Brain Behav, 0:1–16. 10.1080/2153599X.2024.2377545.

[81] Holmberg MJ, Andersen LW. Collider bias. JAMA. 2022;327:1282–3. 10.1001/jama.2022.1820. 35285854 10.1001/jama.2022.1820

[82] Hemani G, Tilling K, Davey Smith G. Orienting the causal relationship between imprecisely measured traits using GWAS summary data. PLoS Genet. 2017;13:e1007081. 10.1371/journal.pgen.1007081. 29149188 10.1371/journal.pgen.1007081 PMC5711033

[83] Sadreev II, Elsworth BL, Mitchell RE, et al. Navigating sample overlap, winner’s curse and weak instrument bias in Mendelian randomization studies using the UK Biobank. medRxiv. 2021. 2021.06.28.21259622. 10.1101/2021.06.28.21259622.

[84] Xue A, Wu Y, Zhu Z, et al. Genome-wide association analyses identify 143 risk variants and putative regulatory mechanisms for type 2 diabetes. Nat Commun. 2018;9:2941. 10.1038/s41467-018-04951-w. 30054458 10.1038/s41467-018-04951-w PMC6063971

[85] Chignon A, Lettre G. Using omics data and genome editing methods to decipher GWAS loci associated with coronary artery disease. Atherosclerosis. 2025;401:118621. 10.1016/j.atherosclerosis.2024.118621. 39909615 10.1016/j.atherosclerosis.2024.118621

[86] Scholtens S, Smidt N, Swertz MA, et al. Cohort profile: lifelines, a three-generation cohort study and biobank. Int J Epidemiol. 2015;44:1172–80. 10.1093/ije/dyu229. 25502107 10.1093/ije/dyu229

[87] Peters A, German National Cohort (NAKO) Consortium, Peters A, et al. Framework and baseline examination of the German National Cohort (NAKO). Eur J Epidemiol. 2022;37:1107–24. 10.1007/s10654-022-00890-5. 36260190 10.1007/s10654-022-00890-5 PMC9581448

[88] Zins M, Bonenfant S, Carton M, et al. The constances cohort: an open epidemiological laboratory. BMC Public Health. 2010;10:479. 10.1186/1471-2458-10-479. 20704723 10.1186/1471-2458-10-479 PMC2927544

[89] Stock J, Yogo M. Testing for Weak Instruments in Linear IV Regression. In: Identification and Inference for Econometric Models. New York: Cambridge University Press; 2005;pp. 80–108. http://www.economics.harvard.edu/faculty/stock/files/TestingWeakInstr_Stock%2BYogo.pdf

[90] Stock JH, Wright JH, Yogo M. A survey of weak instruments and weak identification in generalized method of moments. J Business Econ Stat.200210.1198/073500102288618658.

[91] Olea JLM, Pflueger C. A robust test for weak instruments. J Bus Econ Stat. 2013;31:358–69. 10.1080/00401706.2013.806694.

[92] Sanderson E, Windmeijer F. A weak instrument f-test in linear IV models with multiple endogenous variables. J Econom. 2016;190:212–21. 10.1016/j.jeconom.2015.06.004. 29129953 10.1016/j.jeconom.2015.06.004 PMC5669336

[93] Fadista J, Manning AK, Florez JC, Groop L. The (in)famous GWAS P-value threshold revisited and updated for low-frequency variants. Eur J Hum Genet. 2016;24:1202–5. 10.1038/ejhg.2015.269. 26733288 10.1038/ejhg.2015.269 PMC4970684

[94] Lin Z, Xue H, Pan W. Robust multivariable Mendelian randomization based on constrained maximum likelihood. Am J Hum Genet. 2023;110:592–605. 10.1016/j.ajhg.2023.02.014. 36948188 10.1016/j.ajhg.2023.02.014 PMC10119150

[95] Lorincz-Comi N, Yang Y, Li G, Zhu X. MRBEE: a novel bias-corrected multivariable Mendelian Randomization method. bioRxiv. 2023. 2023.01.10.523480. 10.1101/2023.01.10.523480.

[96] Ye T, Shao J, Kang H. Debiased inverse-variance weighted estimator in two-sample summary-data mendelian randomization. ArXiv. 2020. 10.1002/sim.10245 39453381

[97] Sanderson E, Rosoff D, Palmer T, et al. Bias from heritable confounding in Mendelian randomization studies. medRxiv. 2024. 2024.09.05.24312293. 10.1101/2024.09.05.24312293.

[98] Burgess S, Davies NM, Thompson SG. Instrumental variable analysis with a nonlinear exposure-outcome relationship. Epidemiology. 2014;25:877–85. 10.1097/EDE.0000000000000161. 25166881 10.1097/EDE.0000000000000161 PMC4222800

[99] Staley JR, Burgess S. Semiparametric methods for estimation of a nonlinear exposure-outcome relationship using instrumental variables with application to Mendelian randomization. Genet Epidemiol. 2017;41:341–52. 10.1002/gepi.22041. 28317167 10.1002/gepi.22041 PMC5400068

[100] Wade KH, Hamilton FW, Carslake D, et al. Challenges in undertaking nonlinear Mendelian randomization. Obesity. 2023;31:2887–90. 10.1002/oby.23927. 37845826 10.1002/oby.23927 PMC7615556

[101] Burgess S. Violation of the constant genetic effect assumption can result in biased estimates for non-linear Mendelian randomization. Hum Hered. 2023;88:79–90. 10.1159/000531659. 37651993 10.1159/000531659 PMC10614256

[102] Davey Smith G. Mendelian randomisation and vitamin D: the importance of model assumptions. Lancet Diabetes Endocrinol. 2023;11:14. 10.1016/S2213-8587(22)00345-X. 10.1016/S2213-8587(22)00345-X 36528345

[103] Tian H, Mason AM, Liu C, Burgess S. Relaxing parametric assumptions for non-linear Mendelian randomization using a doubly-ranked stratification method. PLoS Genet. 2023;19:e1010823. 10.1371/journal.pgen.1010823. 37390109 10.1371/journal.pgen.1010823 PMC10343089

[104] Munafò MR, Higgins JPT, Smith GD. Triangulating evidence through the inclusion of genetically informed designs. Cold Spring Harb Perspect Med. 2021;11:a040659. 10.1101/cshperspect.a040659. 33355252 10.1101/cshperspect.a040659 PMC8327826

