# Supplementary material for: Estimating the causal effect of cardiometabolic conditions on socioeconomic and healthcare outcomes: a scoping review of Mendelian randomization studies
Source: Health Econ Rev. 2026 Feb 2;16:23. doi: 10.1186/s13561-026-00724-0 (PMC12930584; doi:10.1186/s13561-026-00724-0)
Supplement: Supplementary file 1 — Additional file 1. [file 13561_2026_724_MOESM1_ESM.docx]

**Estimating the causal effect of cardiometabolic conditions on socio-economic and healthcare outcomes: a scoping review on Mendelian randomization studies**

**Supplementary material**

*Sara Pedron ^a#^, Xiao Tan ^a*#^, Juliane Maushagen^, a,b^, Anna-Janina Stephan ^a^, Jacob Burns ^a^,Eleanor Sanderson ^c, d^, Kaitlin Wade ^c, d^, Michael Laxy^a^*

^a^ Professorship of Public Health and Prevention, School of Medicine and Health, Technical University of Munich, 80992 Munich, Germany

^b^ Institute for Medical Informatics, Biometry and Epidemiology, Ludwig-Maximilians-Universität München, 81377 Munich, Germany

^c^ Population Health Sciences, Bristol Medical School, University of Bristol, Beacon House, Queens Road, Bristol, United Kingdom, BS8 1QU

^d^ Medical Research Council Integrative Epidemiology Unit (MRC-IEU), Oakfield House, Oakfield Grove, Bristol, United Kingdom, BS8 2BN

*^#^* These authors contributed equally

*Corresponding author:

Xiao Tan

[xiao.tan@tum.de](mailto:xiao.tan@tum.de)

ORCID ID: 0009-0009-2979-9841

Appendix 1: Concepts of Mendelian randomization

Appendix 2: PRISMA-ScR checklist

Appendix 3: Search strategy

Appendix 4: Full-text screening results and details on excluded papers

# Appendix 1 Concepts of Mendelian randomization

## Genetic background of Mendelian randomization (MR)

Based on the principles of instrumental variable (IV) analyses, Mendelian randomization is an estimation approach exploiting the randomized genetic variation acquired at conception as instrument [1, 2]. Concretely, MR uses single nucleotide polymorphisms (SNPs) as exogenous sources (or instruments) of variation in the modifiable exposure variable. Nucleotides are components of the genome, which, as a sequence, build the deoxyribonucleic acid (DNA) including the information for the development and functions of organisms. SNPs are common variations of the nucleotide’s base that occur in at least 1% of a population [3]. These variants can cause differences in physiological processes and phenotypes or reflect predispositions for certain diseases, which are investigated in genome-wide association studies (GWASs). According to Mendel’s laws of inheritance, SNPs are randomly inherited at conception and are transmitted independently of individual-level and environmental factors that may otherwise confound the outcome of interest. [4] These two conditions lay the theoretical groundwork for their use as instruments in MR approaches.

## The three core assumptions and potential violations

Since MR is based on IV principles, several assumptions must be valid to allow for causal interpretations of the estimated results. In the following, we describe the three “core” IV assumptions in line with the framework from [5] and illustrate these in Figure S1.

**Figure S1** Graphical depiction of the three core Mendelian randomization assumptions

**
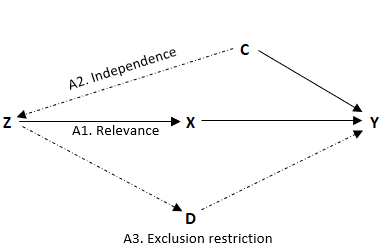
**

X: exposure, Y: outcome, Z: instrument, C: confounders, D: additional pathways. Solid lines are associations that are allowed or desired according to MR assumptions. Dotted lines indicate unwanted associations and suggest violation of the respective assumptions

*Assumption 1: relevance assumption*

According to the relevance assumption, the genetic instrument(s) should have a non-zero average causal effect on the exposure. [5] This assumption will be violated if the proposed instruments are in fact not associated with the exposure, which could lead to an effect overestimation if other IV assumptions (particularly the exclusion restriction) are also violated. [6] If the instrument is significantly but only weakly related with the exposure, weak instrument bias will be induced: this can increase the type 1 error rate and decrease statistical power. [2] As a result, the MR estimates will be biased towards the observational estimates in one-sample MR (MR1) and towards the null in two-sample MR (MR2). [7–9]

To ensure the validity of this assumption, usually only SNPs that achieve genome-wide significance (p < 5x10^-8^) for the investigated exposure are selected in MR studies. Using multiple SNPs or a GRS (also called polygenic risk score - PRS) as an instrument in MR1 or conducting MR2 as sensitivity analyses, which take advantage of the large sample size of the well-powered genetic consortia, are common strategies to circumvent potential weak instrument bias. On the other hand, even for widely studied traits such as body mass index (BMI), the variation explained by available SNPs is often rather small [10], indicating that larger sample size is necessary to reach sufficient statistical power.

*Assumption 2: independence (or exchangeability) assumption*

The independence (or exchangeability) assumption states that instruments are randomly assigned. [5] In other words, there is no confounder of the instrument-outcome correlation. [4, 11] Note that confounding here is defined as a factor that confounds the instrument-outcome association as opposed to the exposure-outcome association, which appears to contradict Mendel’s law of inheritance as genetic variants are set at conception and cannot be affected by any postnatal factors. This holds true for individuals. However, at the population level, there can be mechanisms that influence allele frequencies for the population as described below. As most of the MR studies are based on large GWASs to exploit population causal effects, we choose to use the broader definition of confounding in line with previous MR reviews. [4, 11]

The independence assumption cannot be proved, but it is theoretically plausible in the case of random allocation and independent assignment of SNPs, as the two Mendel´s laws of genetics postulate. [13, 14] However, sources of violation of the independence assumption may exist. [15, 16] In fact, different genetic characteristics within distinct subpopulations (i.e., population stratification), assortative mating, and dynastic effects might be strongly linked with the probability of inheriting certain traits and the investigated outcome, introducing confounding in the effect estimation. Solutions to mitigate these problems include focusing on an ethnically homogeneous population by adjusting for principal components, conducting within-family analyses, and excluding overly related individuals when using population-level data. Note that the observed confounders are usually those of the exposure-outcome associations (e.g., age, sex, education level), which should not have any influence on genetic instruments at the individual level, controlling for them will generally reflect the adjustment for confounder of instrument-outcome associations. However, extensively adjusting for confounding factors could induce collider bias as they can be determined by genetic instruments. Therefore, it is recommended to include only age, sex, principal components, and study centers as control variables in an MR analysis. [12]

*Assumption 3: exclusion restriction*

The exclusion restriction assumes that the effect of the instrument on the outcome is entirely mediated by the exposure of interest. [5] As with the second assumption, this assumption is not testable. In the context of MR studies, there are at least two channels through which this assumption can be questioned: pleiotropy and linkage disequilibrium. [17] Pleiotropy describes a situation in which a single SNP influences multiple traits. [18] When those multiple traits all lie on the same pathway through the exposure towards the outcome (vertical pleiotropy) the exclusion restriction still holds (i.e., no bias is induced). However, when the SNP influences other factors that lie outside the causal pathway via the investigated exposure (horizontal pleiotropy), a violation of exclusion restriction ensues, and the estimates are no longer valid. [19] The second channel of violation, the phenomenon of linkage disequilibrium, refers to the fact that genetic variants in physical proximity are likely to be inherited together. [20] Similar to horizontal pleiotropy, linkage disequilibrium could invalidate the exclusion restriction assumption when the co-inherited genetic variants affect the outcome independent of the exposure.

In the MR context, both horizontal pleiotropy and linkage disequilibrium are serious threats that require substantial understanding of the fundamental and still not well-known genetic mechanisms and functions. Potential solutions involve the use of large public databases [21] to scan for SNPs with known pleiotropic effects or in linkage disequilibrium, the use of MR2 estimators that are robust to pleiotropy, or conducting multivariable MR analysis. [4, 22]

## Further IV assumptions for causal interpretation

Satisfying the three core assumptions is sufficient to determine the presence of a causal effect. To obtain the size of desired effect estimates and to causally interpret them, further assumptions must be valid [5]: homogeneity or monotonicity, and the stable unit treatment value assumption (SUTVA) [23].

*Homogeneity*

In order to interpret the causal estimate as an average causal effect (ACE), the homogeneity assumption must be held. This assumption postulates that either (a) the association between each SNP and the exposure is the same in the whole population or (b) the effect of the exposure on the outcome is the same in the whole population [24]. However, more recently, scholars have shown that weaker assumptions such as the *no effect modification assumption -* that the genetic instrument should not modify the effect of the exposure on the outcome within levels of the exposure and for all levels of the exposure [25] or the *no simultaneous heterogeneity assumption -*that all unmeasured variables that modify the instrument-exposure relation are independent of all unmeasured variables that modify the exposure-outcome relation [26], are also sufficient to assume homogeneity. [27–29]

*Monotonicity*

The monotonicity assumption postulates that the instrument should have a monotonic influence on the exposure for all individuals [5, 30], i.e., the instrument affects all individuals´ phenotype in the same direction. In other words, if we hypothetically change the level of the genetic variants (e.g., more risk-increasing alleles), the level of exposure should change in the same direction (higher odds of being exposed). Under this assumption, a local average treatment effect (LATE) for these individuals can be estimated.

Whether a treatment effect holds for a not definable group of compliers (in case of monotonicity) or for the entire population (in case of homogeneity) is important in the field of health economics, where the primary aim is to quantify population-level economic effects rather than assessing causality per se. Monotonicity or homogeneity cannot be verified; however, while monotonicity seems plausible, consistency of effects of genetic variants with exposures indicates that even homogeneity could hold among European populations. [24, 30]

*SUTVA*

The SUTVA is fundamental for causal inference and requires that the potential outcomes for any unit are independent of treatment assigned to other units (*no interference*), and, across units, there are no different versions of each treatment level (*no hidden variations of treatment*). [5, 31]

In MR analyses, this assumption might be violated since what is estimated corresponds to the effect of the exposure due to genetic liability to a health condition, which might differ from the effect of the same exposure due to environmental or socioeconomic factors. Although scholars have noted that “population genetic analyses of correlations between different traits suggest there are common pathways of genetic and environmental influences” [32], SUTVA is a critical and untestable assumption to be considered in interpreting MR estimates.

*Time horizon*

A specific aspect of interpreting MR estimates is the exposure time. MR estimates can be interpreted as the genetically predicted liability underlying the entire exposure history from conception up to the time the outcome occurs, i.e., the MR estimate represents the effect on the outcome of having a genetic liability for the exposure that results in a one-unit higher level of the exposure, for example, BMI, at the time the exposure and outcome is measured [33]. Only if SNPs have a constant effect on the exposure across the entire lifetime MR estimates can be interpreted as the effect of having a unit higher level of the exposure over the lifetime [34]. This specificity has important implications for how estimates from MR can be used in the context of burden of disease estimations and economic evaluations.

## Methods and conditions for MR estimation

*Individual-level data*

When using individual-level data, that is, when data of genetic instruments, exposures, and outcomes stems from the same study sample (i.e., one-sample MR), MR estimates can usually be obtained using two-stage least squares regression (2SLS).

Let i index individuals and j genetic instruments, the 2SLS can be written as:

(1)

$$Stage 1: X_{ij}=\gamma_{0}+\sum_{j=1}^{J} \gamma_{j}Z_{ij}+\mu_{ij}$$

(2)

$$Stage 2: Y_{i}=\beta_{0}+\beta\hat{X}_{i}+\varepsilon_{i}$$

Where Z is the genetic instrument, X is the exposure, $\hat{X}$ is the estimated exposure from the first stage, Y is the outcome, *β* is the coefficient of interest, and *ε* and *μ* are the error terms. [35, 36]

The genetic instrument can be a SNP, multiple SNPs, or a combined allele risk score (e.g., GRS), which can be unweighted or weighted by the effect size of each SNP on the exposure. When using a single SNP, the 2SLS estimator equals the Wald ratio estimator, which is the ratio of the SNP-outcome association and the SNP-exposure association. When multiple SNPs are available, individual SNPs can be either treated as independent instruments in a 2SLS to fit an over-identified model or they can be combined to a weighted or unweighted GRS to jointly predict the exposure effect on the outcome.

Note that as genetic variants are fixed at conception and cannot be affected by any postnatal factors, covariant adjustment is in principle not needed. Also, controlling for additional covariates in MR studies can induce collider bias as these may be traits that are affected by the proposed genetic variants. [4, 37] However, as mentioned before, difference in allele frequency among population subgroups, dynastic effects, and assortative mating can influence the distribution of genetic variants at the population level, violating the independence assumption. Therefore, it is recommended to incorporate a limited set of covariates such as age, sex, study centers, and principal scores into the MR estimation equations. [11]

Individual-level data enables MR studies to assess the associations between genetic variants and observable confounders of the exposure-outcome relationship to test the independence assumption. It can also be used to assess the associations between genetic variants and environmental factors to test horizontal pleiotropy and/or gene-environmental equivalence. In addition, when multiple instruments are used, Sargan’s overidentification test can be applied to jointly evaluate the independence, exclusion restriction, and homogeneity/monotonicity assumption.

*Summary-level data*

The increasing availability of large-scale GWASs promises the recent proliferation of two-sample MR (MR2) analysis, in which the SNP-exposure and SNP-outcome associations are taken from GWASs with independent study samples, leading to a larger sample size and greater statistical power of MR2. [4, 9] The study samples should represent the same underlying population with no overlapping components, as sample overlap will bias the MR estimates towards the observational ones. [9, 38]

The basic and most efficient analytical approach in a MR2 framework is the inverse-weighted (IVW) method, in which Wald ratio estimator of each SNP is meta-analyzed and combined into a single IVW estimator weighted by the inverse variance of the SNP-outcome association. [39, 40]

Denoting Γ as the total effect of a SNP j on the outcome Y, which comprises of the direct effect α independent of the exposure and the indirect effect β mediated by the exposure, γ as the effect of the SNP j on the exposure, and ε as the error term, the ratio estimator for individual i with SNP j can be written as:

(3)

$$\frac{\Gamma j}{\gamma j}=\frac{\alpha j+\beta\gamma j}{\gamma j}+ \varepsilon i=\frac{\alpha j}{\gamma j}+\beta+ \varepsilon i$$

By combining each ratio estimator using weighted regression, the IVW estimate can be obtained with the following form:

(4)

$$\hat{\beta_{IVW}}=\frac{\sum_{j=1}^{J} {\hat{\gamma_{j}}}^{2}\theta_{Y_{j}}^{-2}\hat{\beta}_{j}}{\sum_{j=1}^{J} \hat{\gamma}_{j}^{2}\theta_{Y_{j}}^{-2}}$$

The variance of the error term in the SNP-Y association models $\theta_{Y_{j}}^{-2}$ can be derived from the source GWAS and represents the weight given for each SNP j. The small variance of the error term in the SNP-exposure association is ignored, requiring no measurement error in the SNP-exposure association. [11]

The IVW method relies on the assumption that there is no horizontal pleiotropy in each SNP or that the pleiotropic effects are balanced out, which means that the α term in equation (3) should be zero for each SNP or it should have an average mean of zero across all SNPs. [39]

A number of pleiotropy-robust methods with less stringent assumptions have been developed und used as sensitivity analyses to assess and correct for horizontal pleiotropy. In Table S1, we summarize the commonly used MR2 sensitivity analyses and their assumptions and refer to [41] and [42] for a more comprehensive overview.

Table S1 Pleiotropy-robust analytical approaches and their assumptions in a two-sample Mendelian randomization framework (adapted and modified from [11])

| **Approach** | **Description** | **Assumptions** | **References** |
| --- | --- | --- | --- |
| MR Egger | Allows for directional pleiotropy  The deviation of MR Eggar intercept from the origin can be used to assess the presence and scope of directional pleiotropy | No Measurement Error (NOME): There is no measurement error in the SNP-exposure association | [41] |
|  |  | Instrument Strength Independent of Direct Effect (InSIDE): The instrument strength should not depend on the direct (pleiotropic) effect of the instrument on the outcome | [42] |
| Median ratio estimator | Robust estimation if at least half of the SNPs are valid | No Measurement Error (NOME): There is no measurement error in the SNP-exposure association | [43] |
|  |  | For simple median approach: no more than 50% of the SNPs have pleiotropic effects  For weighted median approach: no more than 50% of the weight (i.e., an inverse function of the variance of the SNP-outcome associations) stems from SNPs that have pleiotropic effects |  |
| Modal-based estimator | By grouping SNPs into clusters based on their effect similarity, robust estimation can be made if the SNPs in the largest cluster are valid | No Measurement Error (NOME): There is no measurement error in the SNP-exposure association | [44] |
|  |  | Zero Modal Pleiotropy Assumption (ZEMPA): No pleiotropic effect of SNPs in the largest homogeneous cluster, even if the majority of the SNPs are invalid |  |

# Appendix 2 PRISMA-ScR checklist

Preferred Reporting Items for Systematic reviews and Meta-Analyses extension for Scoping Reviews (PRISMA-ScR) Checklist

| **SECTION** | **ITEM** | **PRISMA-ScR CHECKLIST ITEM** | **REPORTED ON PAGE #** |
| --- | --- | --- | --- |
| **TITLE** | | | |
| Title | 1 | Identify the report as a scoping review. | 1 |
| **ABSTRACT** | | | |
| Structured summary | 2 | Provide a structured summary that includes (as applicable): background, objectives, eligibility criteria, sources of evidence, charting methods, results, and conclusions that relate to the review questions and objectives. | 3 |
| **INTRODUCTION** | | | |
| Rationale | 3 | Describe the rationale for the review in the context of what is already known. Explain why the review questions/objectives lend themselves to a scoping review approach. | 4-5, 8 |
| Objectives | 4 | Provide an explicit statement of the questions and objectives being addressed with reference to their key elements (e.g., population or participants, concepts, and context) or other relevant key elements used to conceptualize the review questions and/or objectives. | 5-8, Appendix 1 |
| **METHODS** | | | |
| Protocol and registration | 5 | Indicate whether a review protocol exists; state if and where it can be accessed (e.g., a Web address); and if available, provide registration information, including the registration number. | 8 |
| Eligibility criteria | 6 | Specify characteristics of the sources of evidence used as eligibility criteria (e.g., years considered, language, and publication status), and provide a rationale. | 9-10, Table 1 |
| Information sources* | 7 | Describe all information sources in the search (e.g., databases with dates of coverage and contact with authors to identify additional sources), as well as the date the most recent search was executed. | 8, 10 |
| Search | 8 | Present the full electronic search strategy for at least 1 database, including any limits used, such that it could be repeated. | 10, Appendix 3 |
| Selection of sources of evidence† | 9 | State the process for selecting sources of evidence (i.e., screening and eligibility) included in the scoping review. | 10-11 |
| Data charting process‡ | 10 | Describe the methods of charting data from the included sources of evidence (e.g., calibrated forms or forms that have been tested by the team before their use, and whether data charting was done independently or in duplicate) and any processes for obtaining and confirming data from investigators. | 11 |
| Data items | 11 | List and define all variables for which data were sought and any assumptions and simplifications made. | 11 |
| Critical appraisal of individual sources of evidence | 12 | If done, provide a rationale for conducting a critical appraisal of included sources of evidence; describe the methods used and how this information was used in any data synthesis (if appropriate). | NA (statement provided on page 11 in Manuscript) |
| Synthesis of results | 13 | Describe the methods of handling and summarizing the data that were charted. | 11 |
| **RESULTS** | | | |
| Selection of sources of evidence | 14 | Give numbers of sources of evidence screened, assessed for eligibility, and included in the review, with reasons for exclusions at each stage, ideally using a flow diagram. | 12, Figure 1, Appendix 4 |
| Characteristics of sources of evidence | 15 | For each source of evidence, present characteristics for which data were charted and provide the citations. | 13-15, Figure 2-3, Appendix 5-6 |
| Critical appraisal within sources of evidence | 16 | If done, present data on critical appraisal of included sources of evidence (see item 12). | NA (see item 12) |
| Results of individual sources of evidence | 17 | For each included source of evidence, present the relevant data that were charted that relate to the review questions and objectives. | Appendix 6-7 |
| Synthesis of results | 18 | Summarize and/or present the charting results as they relate to the review questions and objectives. | 15-25, Table 2-6 |
| **DISCUSSION** | | | |
| Summary of evidence | 19 | Summarize the main results (including an overview of concepts, themes, and types of evidence available), link to the review questions and objectives, and consider the relevance to key groups. | 25-30 |
| Limitations | 20 | Discuss the limitations of the scoping review process. | 30 |
| Conclusions | 21 | Provide a general interpretation of the results with respect to the review questions and objectives, as well as potential implications and/or next steps. | 31 |
| **FUNDING** | | | |
| Funding | 22 | Describe sources of funding for the included sources of evidence, as well as sources of funding for the scoping review. Describe the role of the funders of the scoping review. | 2 |

# Appendix 3 Search strategy

For the cardiovascular conditions, we included all relevant MeSH terms under the headings “cardiovascular diseases”, and then specifically “heart diseases” and “vascular diseases”, while excluding any congenital disease, infection, and pregnancy-related cardiovascular complications. We complemented this search by including a wide range of keywords and synonyms for the most common cardiovascular diseases. Similarly, for type 2 diabetes, we included the MeSH term “diabetes, type 2”, and complemented this with related keywords. For the risk factors, we included both the single MeSH terms for any parameter and condition of interest, as well as a range of specific keywords and synonyms. For the definition of MR, we specifically included the MeSH term “Mendelian Randomization Analysis” and related synonyms as keywords.

| # | Query |
| --- | --- |
| 1 | Cardiovascular Diseases/ |
| 2 | heart diseases/ or exp arrhythmias, cardiac/ or exp cardiac conduction system disease/ or exp cardiac output, high/ or exp cardiac output, low/ or exp cardiac tamponade/ or exp cardiomegaly/ or exp cardiomyopathies/ or exp cardiotoxicity/ or exp heart aneurysm/ or exp heart arrest/ or exp heart failure/ or exp heart rupture/ or exp heart valve diseases/ or exp myocardial ischemia/ or exp myocardial stunning/ or exp pericardial effusion/ or exp pneumopericardium/ or exp post-cardiac arrest syndrome/ or exp post pericardiotomy syndrome/ or exp pulmonary heart disease/ or exp rheumatic heart disease/ or exp ventricular dysfunction/ or exp ventricular outflow obstruction/ |
| 3 | vascular diseases/ or exp aneurysm/ or exp angiodysplasia/ or exp angiomatosis/ or exp angioedema/ or exp aortic diseases/ or exp arterial occlusive diseases/ or exp capillary leak syndrome/ or exp cerebrovascular disorders/ or exp colitis, ischemic/ or exp compartment syndromes/ or exp diabetic angiopathies/ or exp "embolism and thrombosis"/ or exp hand-arm vibration syndrome/ or exp hemorrhoids/ or exp hemostatic disorders/ or exp hepatic veno-occlusive disease/ or exp hyperemia/ or exp hypertension/ or exp hypotension/ or exp mesenteric ischemia/ or exp myocardial ischemia/ or exp optic neuropathy, ischemic/ or exp peliosis hepatis/ or exp peripheral vascular diseases/ or exp prehypertension/ or exp pulmonary veno-occlusive disease/ or exp reperfusion injury/ or exp retinal vein occlusion/ or exp scimitar syndrome/ or exp spinal cord vascular diseases/ or exp splenic infarction/ or exp stenosis, pulmonary vein/ or exp superior vena cava syndrome/ or exp telangiectasis/ or exp thoracic outlet syndrome/ or exp varicocele/ or exp varicose veins/ or exp vascular fistula/ or exp vascular system injuries/ or exp vasculitis/ or exp vasoplegia/ or exp venous insufficiency/ |
| 4 | vascular diseases/ or exp aneurysm/ or exp angiodysplasia/ or exp angiomatosis/ or exp angioedema/ or exp aortic diseases/ or exp arterial occlusive diseases/ or exp capillary leak syndrome/ or exp cerebrovascular disorders/ or exp colitis, ischemic/ or exp compartment syndromes/ or exp diabetic angiopathies/ or exp "embolism and thrombosis"/ or exp hand-arm vibration syndrome/ or exp hemorrhoids/ or exp hemostatic disorders/ or exp hepatic veno-occlusive disease/ or exp hyperemia/ or exp hypertension/ or exp hypotension/ or exp mesenteric ischemia/ or exp myocardial ischemia/ or exp optic neuropathy, ischemic/ or exp peliosis hepatis/ or exp peripheral vascular diseases/ or exp prehypertension/ or exp pulmonary veno-occlusive disease/ or exp reperfusion injury/ or exp retinal vein occlusion/ or exp scimitar syndrome/ or exp spinal cord vascular diseases/ or exp splenic infarction/ or exp stenosis, pulmonary vein/ or exp superior vena cava syndrome/ or exp telangiectasis/ or exp thoracic outlet syndrome/ or exp varicocele/ or exp varicose veins/ or exp vascular fistula/ or exp vascular system injuries/ or exp vasculitis/ or exp vasoplegia/ or exp venous insufficiency/ |
| 5 | (cardiovasc* or cardio-vasc* or cerebrovasc* or cerebro-vasc* or cerebral vasc* or cerebral-vasc* or cardiometabolic* or cardio-metabolic*).mp. |
| 6 | ((myocard* or heart* or cardi* or circulat* or coronary) adj3 (infarct* or isch?em* or shock* or attack* or dysfunction* or fail* or insufficien* or decompensat* or arrest* or death* or diseas*)).mp. |
| 7 | ((myocard* or heart* or cardi* or circulat* or coronary) adj3 (infarct* or isch?em* or shock* or attack* or dysfunction* or fail* or insufficien* or decompensat* or arrest* or death* or diseas*)).mp. |
| 8 | (infarct* or isch?mi*).mp. |
| 9 | coronary-artery diseas*.mp. |
| 10 | (angina* or stenocardi* or "angor pectoris").mp. |
| 11 | acute coronary syndr*.mp. |
| 12 | (arrhythm* or dysrhythm* or "atrial fibrillation*" or flutter or "irregular pulse*" or "irregular heart rhythm*" or "abnormal heart rhythm*").mp. |
| 13 | ventricular dysfunction*.mp. |
| 14 | (cardiom#opath* or myocardiopath* or "*carditis").mp. |
| 15 | (cardi* adj2 myopath*).mp. |
| 16 | aneurysm*.mp. |
| 17 | ((stroke* or apople* or cerebral or vasc* or cerebrovasc* or brain* or intracran* or intracerebral) adj5 (isch?emi* or infarct* or thrombo* or emboli* or occlus* or hypoxi* or h?emorrag* or h?ematoma* or bleed* or attack*)).mp. |
| 18 | (stroke* or apople*).mp. |
| 19 | ((arter* or vascular* or vein* or veno* or peripher* or leg* or limb* or (lower adj3 extremit*)) adj3 (occlus* or steno* or obstruct* or lesio* or block* or obliter* or diseas*)).mp. |
| 20 | (dysvascular* or "intermittent claudicat*" or thrombo*).mp. |
| 21 | exp Diabetes Mellitus, Type 2/ |
| 22 | (diabet* or T2DM).mp. |
| 23 | Metabolic Syndrome/ |
| 24 | ("metabolic syndr*" or "dysmetabolic syndr*" or "Reaven syndr*" or "syndr* X" or "insulin-resist* syndr*").mp. |
| 25 | exp Hypertension/ |
| 26 | exp Blood Pressure/ |
| 27 | (hypertens* or "blood press*").mp. |
| 28 | exp Overweight/ |
| 29 | body mass index/ or body size/ or body weight/ or skinfold thickness/ or waist-hip ratio/ |
| 30 | ("body size" or "body weight" or "bodyweight" or "abdominal fat" or "body fat" or "waist hip ratio" or "waist-hip ratio" or "waist-hip-ratio" or "waist to hip ratio" or "waist-to-hip ratio" or "waist-to-hip-ratio" or "waist circumference" or "weight-to-height ratio" or "skinfold thickness" or obes* or adipos* or overweight* or "over weight*").mp. |
| 31 | exp Hyperglycemia/ |
| 32 | exp Insulin Resistance/ |
| 33 | Blood Glucose/ |
| 34 | Glycated Hemoglobin A/ |
| 35 | Prediabetic State/ |
| 36 | ("impaired glucose" adj (toleranc* or stat* or respons* or control* or regul* or homeost* or metab*)).mp. |
| 37 | ("reduced glucose" adj (metab* or toleranc*)).mp. |
| 38 | (("impaired insulin*" or "reduced insulin*") adj secret*).mp. |
| 39 | ("glucose intoleran*" or HbA1c or "glyc* h?emoglobin" or "insulin* resistan*" or "pr?ediabet*" or "pr?e diabet*" or hypergl#c?em*).mp. |
| 40 | ((high or impaired) adj ("fasting glucose" or "blood glucose" or "blood sugar" or "fasting gl#c?emia*")).mp. |
| 41 | exp Dyslipidemias/ |
| 42 | Cholesterol/ |
| 43 | cholesterol, hdl/ or cholesterol, ldl/ |
| 44 | (cholester* or hyperlipid?emia* or hyperlip?emia* or lip?emia* or dyslipid?emia* or hypercholesterol?emia* or hypercholester?emia* or hyperlipoprotein?emia* or LDL or HDL or triglycerid* or hypertriglycerid?emia* or lipoprotein*).mp. |
| 45 | exp Triglycerides/ |
| 46 | exp Lipoproteins/ |
| 47 | exp Arteriosclerosis/ |
| 48 | (atheroscler* or arterioscler* or "arter* scler*").mp. |
| 49 | 1 or 2 or 3 or 4 or 5 or 6 or 7 or 8 or 9 or 10 or 11 or 12 or 13 or 14 or 15 or 16 or 17 or 18 or 19 or 20 or 21 or 22 or 23 or 24 or 25 or 26 or 27 or 28 or 29 or 30 or 31 or 32 or 33 or 34 or 35 or 36 or 37 or 38 or 39 or 40 or 41 or 42 or 43 or 44 or 45 or 46 or 47 or 48 |
| 50 | Mendelian Randomization Analysis/ |
| 51 | ("mendelian randomi?ation" or "gene* instrument*" or "instrument* variable").mp. |
| 52 | 50 or 51 |
| 53 | 49 and 52 |

# Appendix 4 Full-text screening table and detailed information on excluded studies

| Studies | Did the study assess the effect of a cardio-metabolic condition or risk factor? | Was the outcome a measure of individual socio-economic status, healthcare economic outcomes or health utility? | Was the analysis type a Mendelian randomization analysis (i.e., using genetic variants as IVs)? | Was estimating the effect of investigated exposure and outcome the principal aim of this study? | Full-text available? | Include/Exclude | Reason for exclusion |
| --- | --- | --- | --- | --- | --- | --- | --- |
| Böckerman_2019 [47] | Yes | Yes | Yes | Yes | Yes | Include |  |
| Campbell_2021[48] | Yes | Yes | Yes | Yes | Yes | Include |  |
| Dixon_2020[39] | Yes | Yes | Yes | Yes | Yes | Include |  |
| Edwards_2021[49] | Yes | Yes | Yes | Yes | Yes | Include |  |
| Edwards_2022[50] | Yes | Yes | Yes | Yes | Yes | Include |  |
| Harrison_2020[51] | Yes | Yes | Yes | Yes | Yes | Include |  |
| Hazewinkel_2022[52] | Yes | Yes | Yes | Yes | Yes | Include |  |
| Howe_2020[53] | Yes | Yes | Yes | Yes | Yes | Include |  |
| Kurz_2020[54] | Yes | Yes | Yes | Yes | Yes | Include |  |
| Pedron_2021[55] | Yes | Yes | Yes | Yes | Yes | Include |  |
| Tyrrell_2016[56] | Yes | Yes | Yes | Yes | Yes | Include |  |
| Dick_2021[57] | Yes | Yes | Yes | Yes | Yes | Include |  |
| Dixon_2022[58] | Yes | Yes | Yes | Yes | Yes | Include |  |
| Harrison_2021[59] | Yes | Yes | Yes | Yes | Yes | Include |  |
| von Hinke_2012[60] | Yes | Yes | Yes | Yes | Yes | Include |  |
| Hughes_2021[61] | Yes | Yes | Yes | Yes | Yes | Include |  |
| *Wrong exposure/outcome (N=8)* | | | | | | | |
| Tillmann_2017[62] | No | No | Yes | Yes | Yes | Exclude | Wrong exposure/outcome |
| Pehkonen_2021[63] | No | Yes | Yes | Yes | Yes | Exclude | Wrong exposure/outcome |
| Millard_2015[64] | Yes | No | Yes | Yes | Yes | Exclude | Wrong exposure/outcome |
| He_2022[65] | Yes | No | Yes | Yes | Yes | Exclude | Wrong exposure/outcome |
| Ademi_2020[66] | Yes | No | Yes | No | Yes | Exclude | Wrong exposure/outcome |
| van den Broek_2018[67] | Yes | No | Yes | Yes | Yes | Exclude | Wrong exposure/outcome |
| Wootton_2018[68] | Yes | No | Yes | Yes | Yes | Exclude | Wrong exposure/outcome |
| Daghlas_2021[69] | Yes | No | Yes | Yes | Yes | Exclude | Wrong exposure/outcome |
| *Use of a different methodology (N=18)* | | | | | | | |
| Black_2018[70] | Yes | Yes | No | Yes | Yes | Exclude | No use of genetic variants as IVs |
| Cawley_2012[71] | Yes | Yes | No | Yes | Yes | Exclude | No use of genetic variants as IVs |
| Kim_2016[72] | Yes | Yes | No | Yes | Yes | Exclude | No use of genetic variants as IVs |
| Kim_2020[73] | Yes | Yes | No | Yes | Yes | Exclude | No use of genetic variants as IVs |
| Mora_2015[74] | Yes | Yes | No | Yes | Yes | Exclude | No use of genetic variants as IVs |
| Qin_2016[75] | Yes | Yes | No | Yes | Yes | Exclude | No use of genetic variants as IVs |
| Dou_2020[76] | Yes | Yes | No | Yes | Yes | Exclude | No use of genetic variants as IVs |
| Fu_2019[77] | Yes | Yes | No | Yes | Yes | Exclude | No use of genetic variants as IVs |
| Seuring_2015[78] | Yes | Yes | No | Yes | Yes | Exclude | No use of genetic variants as IVs |
| Kinge_2016[79] | Yes | Yes | No | Yes | Yes | Exclude | No use of genetic variants as IVs |
| Kinge_2018[80] | Yes | Yes | No | Yes | Yes | Exclude | No use of genetic variants as IVs |
| Morris_2006[81] | Yes | Yes | No | Yes | Yes | Exclude | No use of genetic variants as IVs |
| Morris_2007[82] | Yes | Yes | No | Yes | Yes | Exclude | No use of genetic variants as IVs |
| Sabia_2007[83] | Yes | Yes | No | Yes | Yes | Exclude | No use of genetic variants as IVs |
| Sabia_2011[84] | Yes | Yes | No | Yes | Yes | Exclude | No use of genetic variants as IVs |
| Sari_2018[85] | Yes | Yes | No | Yes | Yes | Exclude | No use of genetic variants as IVs |
| Barbieri_2022[86] | Yes | Yes | No | Yes | Yes | Exclude | No use of genetic variants as IVs |
| O'Neill_2013[87] | Yes | Yes | No | No | Yes | Exclude | No use of genetic variants as IVs |
| *Seminal papers, genetic variants but no GWAS variants as instruments (N=3)* | | | | | | | |
| Ding_2009[88] | Yes | Yes | No | Yes | Yes | Exclude | No use of GWAS significant SNPs as instruments (candidate gene approach) |
| Norton_2008[89] | Yes | Yes | No | Yes | Yes | Exclude | No use of GWAS significant SNPs as instruments (candidate gene approach) |
| Fletcher_2011[90] | Yes | Yes | No | No | No | Exclude | No use of GWAS significant SNPs as instruments (candidate gene approach) |
| *No IV estimation (N=3)* | | | | | | | |
| Patel_2019[91] | No | Yes | No | Yes | Yes | Exclude | Use of genetic variation, but not as instrument |
| Pehkonen_2019[92] | No | Yes | No | Yes | Yes | Exclude | Use of genetic variation, but not as instrument |
| Alva_2014[93] | Yes | Yes | No | Yes | Yes | Exclude | Use of fixed-effect estimation, no IV |
| *Explanatory/theoretical paper (N=1)* | | | | | | | |
| Dixon_2016[94] | No | No | No | No | Yes | Exclude | No estimation conducted |
| *Phenome-wide association studies (PheWAS) (N=3)* | | | | | | | |
| Dong_2021[95] | Yes | Yes | Yes | No | Yes | Excluded |  |
| Millard_2019[96] | Yes | Yes | Yes | No | Yes | Excluded |  |
| Wang_2022[97] | Yes | Yes | Yes | No | Yes | Excluded |  |
| *MR estimation used as an example for methodological paper (N=3)* | | | | | | | |
| Brumpton_2020[14] | Yes | Yes | Yes | No | Yes | Exclude | Focus on methodological discussion/development |
| Darrous_2021[98] | Yes | Yes | Yes | No | Yes | Exclude | Focus on methodological discussion/development |
| Bao_2019[99] | Yes | Yes | Yes | No | Yes | Exclude | Focus on methodological discussion/development |
| *Abstracts, no full paper available yet (N=4)* | | | | | | | |
| Sulc_2019[100] | Yes | Yes | Yes | Yes | No | Exclude | Abstract, corresponding article not found |
| Sulc_2020[101] | Yes | Yes | Yes | Yes | No | Exclude | Abstract, corresponding article not found |
| Segal_2019[102] | Yes | Yes | Yes | Yes | No | Exclude | Abstract, corresponding article not found |
| Asaria_2013[103] | No | No | No | No | No | Exclude | Abstract, corresponding article not found |

Among the studies screened in the full-text stage, the majority (25 studies) were excluded because they employed a methodology different than MR. Of these, 18 studies [70–87] used variables other than genetic variants as IVs. Furthermore, three studies [91–93] used genetic variation in their estimation but not as an instrument. A further three studies [88–90] that were excluded at this stage represented seminal papers in the use of genetic variation as instruments, using a candidate gene approach. Since the instruments used were not GWAS significant SNPs as in a classic MR analysis, these studies were excluded. One further study [94] presented a theoretical explanation of MR to estimate marginal healthcare costs but did not carry out any analysis and was therefore excluded.

Additionally, eight studies [62–69] focused on exposures or outcomes that did not fit into our inclusion criteria and were therefore excluded. One study [62] carried out a bidirectional MR of the effect of education on coronary heart disease. However, the bidirectional analysis was not the main aim of the study but was used by the authors as a check whether the genetic risk for coronary heart disease was a causal factor for educational attainment, indicating large problems of pleiotropy in their sample. For this reason, the study was excluded.

Furthermore, six studies [14, 95–99] were excluded because their main aim was not to estimate the effect of eligible exposures on included outcomes. Concretely, three studies carried out MR analysis of exposures and outcomes that fit into our inclusion criteria. However, the focus of their study was to try out new methodologies, illustrating them using eligible exposures and outcomes as examples. Further three studies carried out MR phenome-wide association studies (PheWAS), i.e., they estimated large amounts of effects in an automated approach exploiting as many variables as possible in their dataset. For example, two studies [95, 96] estimated the effect of BMI on all potential phenotypes available in their dataset using MR analysis, including 263 and 22,922 outcomes, respectively. Another study [97] tested the effect of 5,048 exposures on risk of atrial fibrillation, and the genetically predicted effects of atrial fibrillation on 10,308 outcomes via two-sample MR analysis. Due to the large number of outcomes included, all studies considered measures that would be eligible according to our inclusion criteria. However, the scope of the studies was to find associations rather than test specific hypotheses, usually neglecting a thorough discussion of assumptions given the large number of outcomes included. Therefore, they were excluded from the final pool of included studies.

Finally, four studies [100–103] were excluded because no corresponding full text was available.

# References

1. Lawlor DA, Harbord RM, Sterne JAC, et al (2008) Mendelian randomization: using genes as instruments for making causal inferences in epidemiology. In: Statistics in Medicine

2. Burgess S, Small DS, Thompson SG (2017) A review of instrumental variable estimators for Mendelian randomization. Stat Methods Med Res 26:2333–2355. https://doi.org/10.1177/0962280215597579

3. von Hinke S, Davey Smith G, Lawlor DA, et al (2016) Genetic markers as instrumental variables. Journal of Health Economics 45:131–148. https://doi.org/10.1016/j.jhealeco.2015.10.007

4. Sanderson E, Glymour MM, Holmes MV, et al (2022) Mendelian randomization. Nat Rev Methods Primers 2:1–21. https://doi.org/10.1038/s43586-021-00092-5

5. Angrist JD, Imbens GW, Rubin DB (2012) Identification of Causal Effects Using Instrumental Variables. Journal of the American Statistical Association

6. Hernán MA, Robins JM (2006) Instruments for Causal Inference: An Epidemiologist’s Dream? Epidemiology 17:360–372. https://doi.org/10.1097/01.ede.0000222409.00878.37

7. Burgess S, Thompson SG, CRP CHD Genetics Collaboration (2011) Avoiding bias from weak instruments in Mendelian randomization studies. International Journal of Epidemiology 40:755–764. https://doi.org/10.1093/ije/dyr036

8. Teumer A (2018) Common Methods for Performing Mendelian Randomization. Front Cardiovasc Med 5:. https://doi.org/10.3389/fcvm.2018.00051

9. Lawlor DA (2016) Commentary: Two-sample Mendelian randomization: opportunities and challenges. Int J Epidemiol 45:908–915. https://doi.org/10.1093/ije/dyw127

10. Loos RJF, Yeo GSH (2022) The genetics of obesity: from discovery to biology. Nat Rev Genet 23:120–133. https://doi.org/10.1038/s41576-021-00414-z

11. Richmond RC, Davey Smith G (2022) Mendelian Randomization: Concepts and Scope. Cold Spring Harb Perspect Med 12:a040501. https://doi.org/10.1101/cshperspect.a040501

12. Bateson W, Mendel G (2009) Mendel’s Principles of Heredity: A Defence, with a Translation of Mendel’s Original Papers on Hybridisation, 1st ed. Cambridge University Press

13. Sheehan NA, Didelez V, Burton PR, Tobin MD (2008) Mendelian Randomisation and Causal Inference in Observational Epidemiology. PLoS Med 5:e177. https://doi.org/10.1371/journal.pmed.0050177

14. Brumpton B, Sanderson E, Heilbron K, et al (2020) Avoiding dynastic, assortative mating, and population stratification biases in Mendelian randomization through within-family analyses. Nat Commun 11:3519. https://doi.org/10.1038/s41467-020-17117-4

15. Goodarzi MO (2018) Genetics of obesity: what genetic association studies have taught us about the biology of obesity and its complications. The Lancet Diabetes & Endocrinology 6:223–236. https://doi.org/10.1016/S2213-8587(17)30200-0

16. Burgess S, Davey Smith G, Davies NM, et al (2023) Guidelines for performing Mendelian randomization investigations: update for summer 2023. Wellcome Open Res 4:186. https://doi.org/10.12688/wellcomeopenres.15555.3

17. Smith GD, Hemani G (2014) Mendelian randomization: genetic anchors for causal inference in epidemiological studies. Hum Mol Genet 23:. https://doi.org/10.1093/hmg/ddu328

18. Hu JX, Thomas CE, Brunak S (2016) Network biology concepts in complex disease comorbidities. Nat Rev Genet 17:615–629. https://doi.org/10.1038/nrg.2016.87

19. Pingault J-B, O’Reilly PF, Schoeler T, et al (2018) Using genetic data to strengthen causal inference in observational research. Nat Rev Genet 19:566–580. https://doi.org/10.1038/s41576-018-0020-3

20. Solovieff N, Cotsapas C, Lee PH, et al (2013) Pleiotropy in complex traits: challenges and strategies. Nat Rev Genet 14:483–495. https://doi.org/10.1038/nrg3461

21. Staley JR, Blackshaw J, Kamat MA, et al (2016) PhenoScanner: a database of human genotype–phenotype associations. Bioinformatics 32:3207–3209. https://doi.org/10.1093/bioinformatics/btw373

22. Sanderson E, Davey Smith G, Windmeijer F, Bowden J (2019) An examination of multivariable Mendelian randomization in the single-sample and two-sample summary data settings. International Journal of Epidemiology 48:713–727. https://doi.org/10.1093/ije/dyy262

23. Labrecque J, Swanson SA (2018) Understanding the Assumptions Underlying Instrumental Variable Analyses: a Brief Review of Falsification Strategies and Related Tools. Curr Epidemiol Rep 5:214–220. https://doi.org/10.1007/s40471-018-0152-1

24. MR Dictionary (2022) Homogeneity Assumption. https://mr-dictionary.mrcieu.ac.uk/term/homogeneity/

25. MR Dictionary (2022) No effect modification assumption (Additional IV assumption). https://mr-dictionary.mrcieu.ac.uk/term/no-effect-mod/

26. Hartwig FP, Wang L, Davey Smith G, Davies NM (2023) Average Causal Effect Estimation Via Instrumental Variables: the No Simultaneous Heterogeneity Assumption. Epidemiology 34:325–332. https://doi.org/10.1097/EDE.0000000000001596

27. Sheehan NA, Didelez V (2020) Epidemiology, genetic epidemiology and Mendelian randomisation: more need than ever to attend to detail. Hum Genet 139:121–136. https://doi.org/10.1007/s00439-019-02027-3

28. Small DS, Tan Z, Ramsahai RR, et al (2017) Instrumental Variable Estimation with a Stochastic Monotonicity Assumption. Statistical Science 32:561–579. https://doi.org/10.1214/17-STS623

29. Swanson SA, Hernán MA (2018) The challenging interpretation of instrumental variable estimates under monotonicity. International Journal of Epidemiology 47:1289–1297. https://doi.org/10.1093/ije/dyx038

30. MR Dictionary (2022) Monotonicity Assumption. https://mr-dictionary.mrcieu.ac.uk/term/monotonicity/

31. Imbens GW, Rubin DB (2015) Causal Inference for Statistics, Social, and Biomedical Sciences: An Introduction. Cambridge University Press, Cambridge

32. Ebrahim S, Davey Smith G (2008) Mendelian randomization: can genetic epidemiology help redress the failures of observational epidemiology? Hum Genet 123:15–33. https://doi.org/10.1007/s00439-007-0448-6

33. Morris TT, Heron J, Sanderson ECM, et al (2022) Interpretation of Mendelian randomization using a single measure of an exposure that varies over time. Int J Epidemiol 51:1899–1909. https://doi.org/10.1093/ije/dyac136

34. Labrecque JA, Swanson SA (2019) Interpretation and Potential Biases of Mendelian Randomization Estimates With Time-Varying Exposures. Am J Epidemiol 188:231–238. https://doi.org/10.1093/aje/kwy204

35. Angrist JD, Imbens GW (1995) Two-Stage Least Squares Estimation of Average Causal Effects in Models with Variable Treatment Intensity. Journal of the American Statistical Association 90:431–442. https://doi.org/10.1080/01621459.1995.10476535

36. Wooldridge JM (2010) Econometric analysis of cross section and panel data. MIT press

37. Munafò MR, Tilling K, Taylor AE, et al (2018) Collider scope: when selection bias can substantially influence observed associations. Int J Epidemiol 47:226–235. https://doi.org/10.1093/ije/dyx206

38. Burgess S, Davies NM, Thompson SG (2016) Bias due to participant overlap in two-sample Mendelian randomization. Genetic Epidemiology 40:597–608. https://doi.org/10.1002/gepi.21998

39. Dixon P, Hollingworth W, Harrison S, et al (2020) Mendelian Randomization analysis of the causal effect of adiposity on hospital costs. Journal of Health Economics 70:102300. https://doi.org/10.1016/J.JHEALECO.2020.102300

40. Burgess S, Butterworth A, Thompson SG (2013) Mendelian Randomization Analysis With Multiple Genetic Variants Using Summarized Data. Genetic Epidemiology 37:658–665. https://doi.org/10.1002/gepi.21758

41. Mbutiwi FIN, Dessy T, Sylvestre M-P (2022) Mendelian Randomization: A Review of Methods for the Prevention, Assessment, and Discussion of Pleiotropy in Studies Using the Fat Mass and Obesity-Associated Gene as an Instrument for Adiposity. Front Genet 13:803238. https://doi.org/10.3389/fgene.2022.803238

42. Boehm FJ, Zhou X (2022) Statistical methods for Mendelian randomization in genome-wide association studies: A review. Computational and Structural Biotechnology Journal 20:2338–2351. https://doi.org/10.1016/j.csbj.2022.05.015

43. Bowden J, Del Greco M F, Minelli C, et al (2016) Assessing the suitability of summary data for two-sample Mendelian randomization analyses using MR-Egger regression: the role of the I2 statistic. Int J Epidemiol 45:1961–1974. https://doi.org/10.1093/ije/dyw220

44. Bowden J, Davey Smith G, Burgess S (2015) Mendelian randomization with invalid instruments: effect estimation and bias detection through Egger regression. Int J Epidemiol 44:512–525. https://doi.org/10.1093/ije/dyv080

45. Bowden J, Davey Smith G, Haycock PC, Burgess S (2016) Consistent Estimation in Mendelian Randomization with Some Invalid Instruments Using a Weighted Median Estimator. Genet Epidemiol 40:304–314. https://doi.org/10.1002/gepi.21965

46. Hartwig FP, Davey Smith G, Bowden J (2017) Robust inference in summary data Mendelian randomization via the zero modal pleiotropy assumption. Int J Epidemiol 46:1985–1998. https://doi.org/10.1093/ije/dyx102

47. Böckerman P, Cawley J, Viinikainen J, et al (2019) The effect of weight on labor market outcomes: An application of genetic instrumental variables. Health Economics 28:65–77. https://doi.org/10.1002/hec.3828

48. Campbell DD, Green M, Davies N, et al (2021) Effects of increased body mass index on employment status: a Mendelian randomisation study. Int J Obes 45:1790–1801. https://doi.org/10.1038/s41366-021-00846-x

49. Edwards CH, Bjørngaard JH, Minet Kinge J (2021) The relationship between body mass index and income: Using genetic variants from HUNT as instrumental variables. Health Economics 30:1933–1949. https://doi.org/10.1002/hec.4285

50. Edwards CH, Vie GÅ, Kinge JM (2022) Body mass index and healthcare costs: using genetic variants from the HUNT study as instrumental variables. BMC Health Serv Res 22:396. https://doi.org/10.1186/s12913-022-07597-z

51. Harrison S, Davies AR, Dickson M, et al (2020) The causal effects of health conditions and risk factors on social and socioeconomic outcomes: Mendelian randomization in UK Biobank. International Journal of Epidemiology 49:1661–1681. https://doi.org/10.1093/ije/dyaa114

52. Hazewinkel A-D, Richmond RC, Wade KH, Dixon P (2022) Mendelian randomization analysis of the causal impact of body mass index and waist-hip ratio on rates of hospital admission. Economics & Human Biology 44:101088. https://doi.org/10.1016/j.ehb.2021.101088

53. Howe LD, Kanayalal R, Harrison S, et al (2020) Effects of body mass index on relationship status, social contact and socio-economic position: Mendelian randomization and within-sibling study in UK Biobank. International Journal of Epidemiology 49:1173–1184. https://doi.org/10.1093/ije/dyz240

54. Kurz CF, Laxy M (2020) Application of Mendelian Randomization to Investigate the Association of Body Mass Index with Health Care Costs. Med Decis Making 40:156–169. https://doi.org/10.1177/0272989X20905809

55. Pedron S, Kurz CF, Schwettmann L, Laxy M (2021) The Effect of BMI and Type 2 Diabetes on Socioeconomic Status: A Two-Sample Multivariable Mendelian Randomization Study. Diabetes Care 44:850–852. https://doi.org/10.2337/dc20-1721

56. Tyrrell J, Jones SE, Beaumont R, et al (2016) Height, body mass index, and socioeconomic status: mendelian randomisation study in UK Biobank. BMJ i582. https://doi.org/10.1136/bmj.i582

57. Dick K, Schneider JE, Briggs A, et al (2021) Mendelian randomization: estimation of inpatient hospital costs attributable to obesity. Health Econ Rev 11:16. https://doi.org/10.1186/s13561-021-00314-2

58. Dixon P, Harrison S, Hollingworth W, et al (2022) Estimating the causal effect of liability to disease on healthcare costs using Mendelian Randomization. Economics & Human Biology 46:101154. https://doi.org/10.1016/j.ehb.2022.101154

59. Harrison S, Dixon P, Jones HE, et al (2021) Long-term cost-effectiveness of interventions for obesity: A mendelian randomisation study. PLoS Med 18:e1003725. https://doi.org/10.1371/journal.pmed.1003725

60. Von Hinke Kessler Scholder S, Davey Smith G, Lawlor DA, et al (2012) The effect of fat mass on educational attainment: Examining the sensitivity to different identification strategies. Economics & Human Biology 10:405–418. https://doi.org/10.1016/j.ehb.2012.04.015

61. Hughes A, Wade KH, Dickson M, et al (2021) Common health conditions in childhood and adolescence, school absence, and educational attainment: Mendelian randomization study. npj Sci Learn 6:1. https://doi.org/10.1038/s41539-020-00080-6

62. Tillmann T, Vaucher J, Okbay A, et al (2017) Education and coronary heart disease: mendelian randomisation study. BMJ j3542. https://doi.org/10.1136/bmj.j3542

63. Pehkonen J, Viinikainen J, Kari JT, et al (2021) Birth weight and adult income: An examination of mediation through adult height and body mass. Health Economics 30:2383–2398. https://doi.org/10.1002/hec.4387

64. Millard LAC, Davies NM, Timpson NJ, et al (2015) MR-PheWAS: hypothesis prioritization among potential causal effects of body mass index on many outcomes, using Mendelian randomization. Sci Rep 5:16645. https://doi.org/10.1038/srep16645

65. He C, Zhang M, Li J, et al (2022) Novel insights into the consequences of obesity: a phenotype-wide Mendelian randomization study. Eur J Hum Genet 30:540–546. https://doi.org/10.1038/s41431-021-00978-8

66. Ademi Z, Norman R, Pang J, et al (2020) Health economic evaluation of screening and treating children with familial hypercholesterolemia early in life: Many happy returns on investment? Atherosclerosis 304:1–8. https://doi.org/10.1016/j.atherosclerosis.2020.05.007

67. Van Den Broek N, Treur JL, Larsen JK, et al (2018) Causal associations between body mass index and mental health: a Mendelian randomisation study. J Epidemiol Community Health 72:708–710. https://doi.org/10.1136/jech-2017-210000

68. Wootton RE, Lawn RB, Millard LAC, et al (2018) Evaluation of the causal effects between subjective wellbeing and cardiometabolic health: mendelian randomisation study. BMJ k3788. https://doi.org/10.1136/bmj.k3788

69. Daghlas I, Richmond RC, Lane JM, et al (2021) Selection into shift work is influenced by educational attainment and body mass index: a Mendelian randomization study in the UK Biobank. International Journal of Epidemiology 50:1229–1240. https://doi.org/10.1093/ije/dyab031

70. Black N, Hughes R, Jones AM (2018) The health care costs of childhood obesity in Australia: An instrumental variables approach. Economics & Human Biology 31:1–13. https://doi.org/10.1016/j.ehb.2018.07.003

71. Cawley J, Meyerhoefer C (2012) The medical care costs of obesity: An instrumental variables approach. Journal of Health Economics 31:219–230. https://doi.org/10.1016/j.jhealeco.2011.10.003

72. Kim TH, Lee E-K, Han E (2016) Incremental impact of body mass status with modifiable unhealthy lifestyle behaviors on pharmaceutical expenditure. Research in Social and Administrative Pharmacy 12:990–1003. https://doi.org/10.1016/j.sapharm.2015.12.009

73. Kim J-H, Lee W-Y, Lim SS, et al (2020) Gender Differences in the Relationship between Type 2 Diabetes Mellitus and Employment: Evidence from the Korea Health Panel Study. IJERPH 17:7040. https://doi.org/10.3390/ijerph17197040

74. Mora T, Gil J, Sicras-Mainar A (2015) The influence of obesity and overweight on medical costs: a panel data perspective. Eur J Health Econ 16:161–173. https://doi.org/10.1007/s10198-014-0562-z

75. Qin X, Pan J (2016) The Medical Cost Attributable to Obesity and Overweight in China: Estimation Based on Longitudinal Surveys. Health Economics 25:1291–1311. https://doi.org/10.1002/hec.3217

76. Dou J, Du L, Wang K, et al (2020) Wage Penalties or Wage Premiums? A Socioeconomic Analysis of Gender Disparity in Obesity in Urban China. IJERPH 17:7004. https://doi.org/10.3390/ijerph17197004

77. Fu R, Noguchi H, Kaneko S, et al (2019) How do cardiovascular diseases harm labor force participation? Evidence of nationally representative survey data from Japan, a super-aged society. PLoS ONE 14:e0219149. https://doi.org/10.1371/journal.pone.0219149

78. Seuring T, Goryakin Y, Suhrcke M (2015) The impact of diabetes on employment in Mexico. Economics & Human Biology 18:85–100. https://doi.org/10.1016/J.EHB.2015.04.002

79. Kinge JM (2016) Body mass index and employment status: A new look. Economics & Human Biology 22:117–125. https://doi.org/10.1016/j.ehb.2016.03.008

80. Kinge JM, Morris S (2018) The Impact of Childhood Obesity on Health and Health Service Use. Health Serv Res 53:1621–1643. https://doi.org/10.1111/1475-6773.12708

81. Morris S (2006) Body mass index and occupational attainment. Journal of Health Economics 25:347–364. https://doi.org/10.1016/j.jhealeco.2005.09.005

82. Morris S (2007) The impact of obesity on employment. Labour Economics 14:413–433. https://doi.org/10.1016/j.labeco.2006.02.008

83. Sabia JJ (2007) The Effect of Body Weight on Adolescent Academic Performance. Southern Economic Journal 73:871–900. https://doi.org/10.1002/j.2325-8012.2007.tb00809.x

84. Sabia JJ, Rees DI (2011) Body weight and wages: Evidence from Add Health. Economics & Human Biology S1570677X11000955. https://doi.org/10.1016/j.ehb.2011.09.004

85. Sari N, Acan Osman B (2018) The effect of body weight on employment among Canadian women: evidence from Canadian data. Can J Public Health 109:873–881. https://doi.org/10.17269/s41997-018-0097-7

86. Barbieri PN, Nguyen H (2022) Diabetes and Young Adults’ Labor Supply: Evidence from a Novel Instrumental Variable Strategy. J Labor Res 43:1–23. https://doi.org/10.1007/s12122-022-09328-z

87. O’Neill D, Sweetman O (2013) The consequences of measurement error when estimating the impact of obesity on income. IZA J Labor Econ 2:3. https://doi.org/10.1186/2193-8997-2-3

88. Ding W, Lehrer SF, Rosenquist JN, Audrain-McGovern J (2009) The impact of poor health on academic performance: New evidence using genetic markers. Journal of Health Economics 28:578–597. https://doi.org/10.1016/j.jhealeco.2008.11.006

89. Norton EC, Han E (2008) Genetic information, obesity, and labor market outcomes. Health Economics 17:1089–1104. https://doi.org/10.1002/hec.1383

90. Fletcher JM, Lehrer SF (2011) Genetic lotteries within families. Journal of Health Economics 30:647–659. https://doi.org/10.1016/j.jhealeco.2011.04.005

91. Patel PC, Rietveld CA (2019) Effect of Genetic Propensity for Obesity on Income and Wealth Through Educational Attainment. Obesity 27:1423–1427. https://doi.org/10.1002/oby.22528

92. Pehkonen J, Viinikainen J, Böckerman P, et al (2019) Health endowment and later-life outcomes in the labour market: Evidence using genetic risk scores and reduced-form models. SSM - Population Health 7:100379. https://doi.org/10.1016/j.ssmph.2019.100379

93. Alva M, Gray A, Mihaylova B, Clarke P (2014) The effect of diabetes complications on health‐related quality of life: the importance of longitudinal data to address patient heterogeneity. Health Economics 23:487–500. https://doi.org/10.1002/hec.2930

94. Dixon P, Davey Smith G, Von Hinke S, et al (2016) Estimating Marginal Healthcare Costs Using Genetic Variants as Instrumental Variables: Mendelian Randomization in Economic Evaluation. PharmacoEconomics 34:1075–1086. https://doi.org/10.1007/s40273-016-0432-x

95. Dong S-S, Zhang K, Guo Y, et al (2021) Phenome-wide investigation of the causal associations between childhood BMI and adult trait outcomes: a two-sample Mendelian randomization study. Genome Med 13:48. https://doi.org/10.1186/s13073-021-00865-3

96. Millard LAC, Davies NM, Tilling K, et al (2019) Searching for the causal effects of body mass index in over 300 000 participants in UK Biobank, using Mendelian randomization. PLoS Genet 15:e1007951. https://doi.org/10.1371/journal.pgen.1007951

97. Wang Q, Richardson TG, Sanderson E, et al (2022) A phenome-wide bidirectional Mendelian randomization analysis of atrial fibrillation. International Journal of Epidemiology 51:1153–1166. https://doi.org/10.1093/ije/dyac041

98. Darrous L, Mounier N, Kutalik Z (2021) Simultaneous estimation of bi-directional causal effects and heritable confounding from GWAS summary statistics. Nat Commun 12:7274. https://doi.org/10.1038/s41467-021-26970-w

99. Bao Y, Clarke PS, Smart M, Kumari M (2019) Assessing the robustness of sisVIVE in a Mendelian randomization study to estimate the causal effect of body mass index on income using multiple SNPs from understanding society. Statistics in Medicine 38:1529–1542. https://doi.org/10.1002/sim.8066

100. Sulc J, Sonrel A, Kutalik Z (2019) Components of Obesity: Genetic Architecture, Causes, and Consequences - 47th European Mathematical Genetics Meeting (EMGM) 2019. Hum Hered 83:225–249. https://doi.org/10.1159/000499459

101. Sulc J, Sonrel A, Kutalik Z (2020) Heterogeneity in obesity and its consequences on health - The 2020 Annual Meeting of the International Genetic Epidemiology Society. Genetic Epidemiology 44:469–533. https://doi.org/10.1002/gepi.22298

102. Segal AB, Huerta MC, Sassi F (2019) Understanding the effect of childhood obesity and overweight on educational outcomes: an interdisciplinary secondary analysis of two UK cohorts. The Lancet 394:S84. https://doi.org/10.1016/S0140-6736(19)32881-8

103. Asaria M, Walker S, Sculpher MJ, et al (2013) Challenges of Conducting Economic Evaluations Using Linked Electronic Health Records - CPRD and HES in the United Kingdom. Value in Health 16:A580. https://doi.org/10.1016/j.jval.2013.08.1584
